# Messaging Modality and Content for Recruitment of Research Participants: A Randomized Clinical Trial

**DOI:** 10.1001/jamanetworkopen.2026.14046

**Published:** 2026-05-22

**Authors:** Pishoy Gouda, LáShauntá Glover, Aarti Kenjale, Karen Chiswell, Tyler Erickson, Benjamin Goldstein, Michelle Kelsey, Jamie Roberts, Eric D. Peterson, Manesh Patel, George Truskey, Svati Shah, Pamela S. Douglas, Adam J. Nelson, Neha J. Pagidipati

**Affiliations:** 1 Duke Clinical Research Institute, Durham, North Carolina; 2 University of Alberta, Edmonton, Alberta, Canada; 3 Department of Population Health Sciences, Duke University School of Medicine, Durham, North Carolina; 4 Department of Medicine, Duke University, Durham, North Carolina; 5 Duke University Medical Center, Durham, North Carolina; 6 Southwestern Medical School, University of Texas, Dallas

## Abstract

**Importance:**

Optimizing participant recruitment is critical to the success and generalizability of clinical research, yet there is limited randomized clinical trial evidence comparing the effects of digital recruitment strategies and message framing on participant engagement.

**Objective:**

To determine the impact of various recruitment strategies on engagement with potential research participants.

**Design, Setting, and Participants:**

This randomized clinical trial was embedded within the Research for Personalized Cardiovascular Disease Prevention (RESILIENCE) study and had a 2 × 2 factorial design. This component of the RESILIENCE study was exclusively virtual and completed online within a single large academic health system in the United States. Potential research participants were identified through electronic health records. The study recruited participants between September 2019 and March 2022, with the analysis undertaken in July to December 2025. No follow-up was undertaken for this study.

**Interventions:**

Participants were randomized to a recruitment modality (email vs patient portal) and to a type of message content (altruistic vs individualistic).

**Main Outcomes and Measures:**

The primary outcome was participant interest, defined as logging onto the study website by clicking the link in the message within 6 months. A robust log-linear Poisson model was used to estimate the relative risk (RR) of the primary outcome based on modality and message content.

**Results:**

Of 15 376 potential research participants (9309 [60.5%] female) identified through the electronic health records, 7737 (50.3%) were randomized to the email recruitment modality and 7639 (49.7%) were randomized to the patient portal modality; 7682 (50.0%) received an altruistic recruitment message and 7694 (50.0%) received an individualistic recruitment message. The primary outcome occurred in 1220 participants (7.9%). The email recruitment modality led to a higher likelihood of the primary outcome than patient portal messages (768 [9.9%] vs 452 [5.9%]; RR, 1.68; 99% CI, 1.45-1.95), while no difference was observed by message content type (634 [8.3%] altruistic vs 586 [7.6%] individualistic; RR, 1.08; 99% CI, 0.94-1.25). There was no significant interaction between message modality and content. Exploratory interactions by age and risk phenotype were observed, with altruistic messaging more effective than individualistic messaging in those aged 60 years and younger and email more effective than patient portal in those older than 60 years and in individuals with obesity.

**Conclusions and Relevance:**

In this randomized clinical trial of recruitment strategies, email messaging led to higher participant interest than patient portal messaging, particularly in older individuals. Message content did not significantly impact engagement overall, but altruistic messaging was more effective in younger participants. These results can inform recruitment strategies for future trials.

**Trial Registration:**

ClinicalTrials.gov Identifier: NCT04551872

## Key Points

**Question:**

Among adults identified through electronic health records for potential research participation, how do recruitment modality (email vs patient portal) and message content (altruistic vs individualistic) affect participant engagement?

**Findings:**

In this randomized clinical trial of 15 376 potential research participants, email recruitment was associated with significantly higher engagement compared with patient portal messaging. Message content did not significantly affect engagement overall, although exploratory analyses suggested higher engagement with altruistic messaging among participants 60 years and younger.

**Meaning:**

The findings of this study suggest that recruitment modality plays a key role in research engagement, with email outperforming patient portal messaging.

## Teaser

This randomized clinical trial evaluates whether receiving research recruitment information via email vs patient portal message and with altruistic vs individualistic content creates more interest among potential trial participants.

## Introduction

Evidence-based medicine relies on robust studies that are generalizable to the population of interest. However, participant recruitment is one of the most challenging and resource-consuming aspects of a clinical trial.^1^ Nearly half of all studies fail to achieve 80% of their planned sample size, resulting in an inability to interpret the results meaningfully.^2,3^ An equally important issue is that clinical trials have struggled to recruit diverse study populations, with underrecruitment of people who are part of racial or ethnic minority groups, women, and rural-dwelling adults.^4,5,6^ Despite this challenge, recruitment and engagement strategies remain understudied.

With increasing use of email (approximately 90% of US adults use email) and electronic health record (EHR) patient portals (approximately 40% of US adults use patient portals), these modalities are increasingly used for research recruitment.^7^ Studies comparing email and postal mail recruitment have yielded mixed results, ranging from email being less effective to more than 3 times more effective.^8,9^ Similarly, studies comparing patient portal with telephone or postal mail recruitment have suggested that portal-based recruitment is feasible^10,11,12,13^ and may be more cost-effective.^14^ However, data on a head-to-head comparison of email vs patient portal recruitment are lacking.

Equally important to how potential research participants are contacted is the content of the message itself. Messaging content can be crafted to appeal to various motivations (altruistic or individualistic).^15,16^ While many trials do attempt to cater the messaging in recruitment material to their population,^17^ limited research assessed the effectiveness of these strategies. To further examine these strategies, the Research for Personalized Cardiovascular Disease Prevention (RESILIENCE) cohort study embedded a randomized 2 × 2 factorial design into its recruitment process exploring the impact of contact modality and recruitment messages on potential participant engagement.

## Methods

### The RESILIENCE Study

The RESILIENCE study was a prospective study within the Duke University Health System evaluating clinical, behavioral, and biological factors associated with cardiovascular risk and obesity resilience following a 6-month remote behavioral intervention. The study aimed to enroll 600 adults aged 40 to 70 years across 4 prespecified phenotypes defined by body mass index (BMI; calculated as weight in kilograms divided by height in meters squared) and 10-year atherosclerotic cardiovascular disease (ASCVD) risk: phenotype 1, BMI of 30 or greater and ASCVD risk of 20% or greater; phenotype 2, BMI of 30 or greater and ASCVD risk of 7.5% or less; phenotype 3, BMI of 18 to 25 and ASCVD risk of 20% or greater; and phenotype 4, BMI of 18 to 25 and ASCVD risk of 7.5% or less.

Eligible participants required prior clinical engagement within the health system, recorded BMI, a primary care practitioner, and documented electronic contact information. Individuals were excluded if they had opted out of research contact, were pregnant or less than 12 months post partum, or had prior bariatric surgery or known ASCVD.

Potential participants were identified using EHR databased on the eligibility criteria between September 2019 and March 2022. ASCVD risk was calculated using the Pooled Cohort Equation, which includes age, sex, total cholesterol level, high-density lipoprotein cholesterol level, systolic blood pressure, treatment of hypertension, diabetes, and smoking.^18^ In the event of missing data required to calculate ASCVD risk, the worst- and best-case scenarios were imputed using the 1% and 99% percentile values. If using the worst-case scenario, a participant was low risk, then they were categorized as such. Similarly, if using the best-case scenario, a participant was high risk, then they were categorized as such. If categorization was not possible based on these rules, those participants were excluded.

### Design of the Randomized Clinical Trial of Recruitment

**Figure 1.**
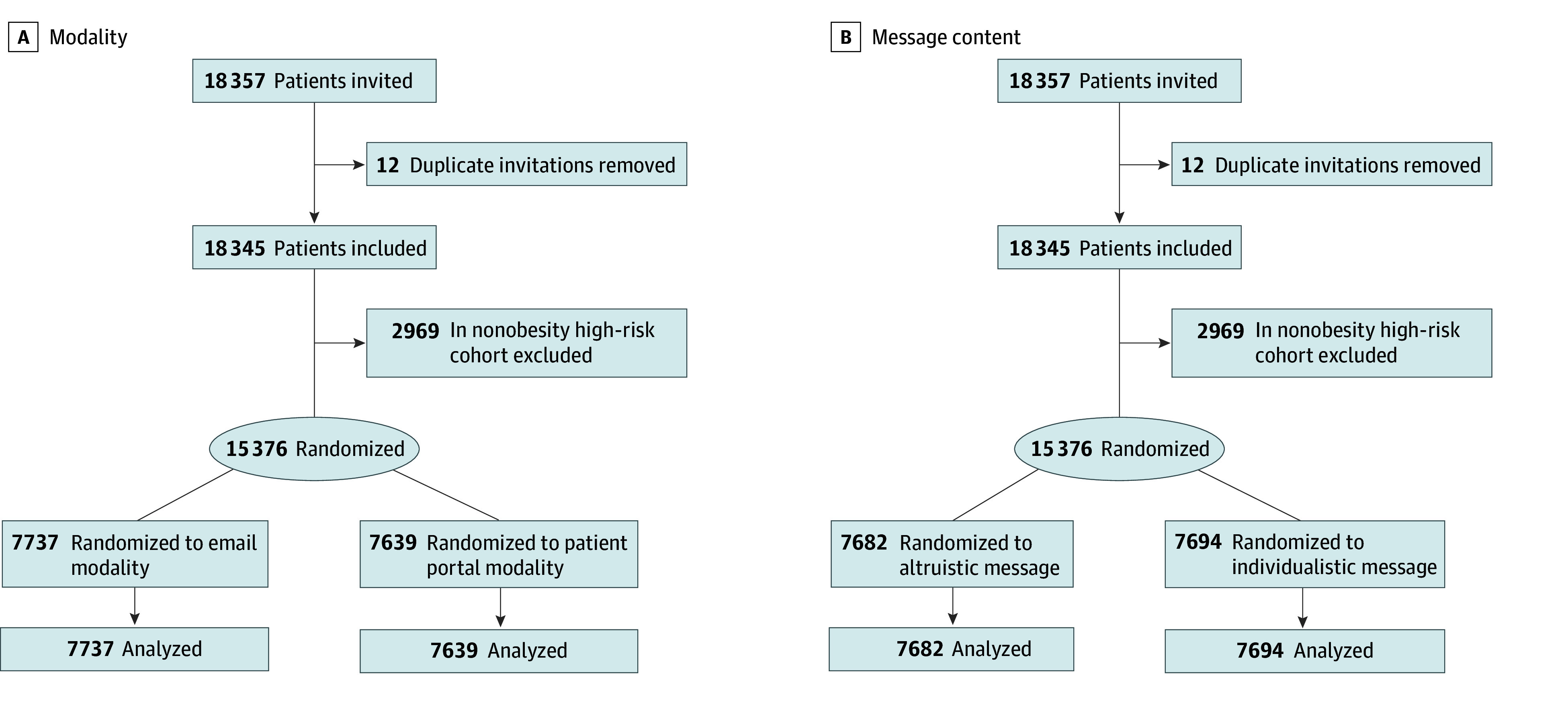
Flowchart of Study

Potentially eligible participants for the RESILIENCE study who met these criteria constituted the pool of participants for an embedded assessment of various recruitment strategies. The recruitment study design was a factorial 2 × 2 randomized clinical trial of (1) method of potential participant contact and (2) content of recruitment message (Figure 1). The RESILIENCE study received approval from the Duke Institutional Review Board. A waiver of a priori consent was granted for the randomized recruitment portion of the study due minimal risk of the study and the lack of feasibility of obtaining consent for a study examining elements of recruitment effectiveness. However, participants who had previously opted out of being contacted for research purposes were not contacted. When patients landed on the study website, they were given additional information about the RESILIENCE study and asked to undergo informed consent for the overall study. The trial protocol and statistical analysis plan appear in Supplement 1. This study is reported as per Consolidated Standards of Reporting Trials (CONSORT) reporting guideline and Studies Within a Trial (SWAT) reporting guidelines.^19,20^

Possible participants were randomized with a 1:1:1:1 allocation to either (1) receive an email from a study-specific email address or through the MyChart patient portal and (2) receive either an altruistic recruitment message or an individualistic message (eTable 1 in Supplement 2). The individualistic message necessarily differed by phenotype to maintain relevance (personal health benefit for participants with obesity and contribution-to-science framing for normal-weight participants), which may limit the conceptual purity of the altruistic vs individualistic contrast. Participants received up to 3 reminders, with a maximum of 1 per week. The content of these messages was crafted in consultation with a longitudinal patient advisory group. The patient advisory group consisted of 4 to 6 individuals with lived experiences relevant to the study and met 5 times during the study period.

Patients were block-randomized stratified on the 4 phenotypes identified previously. The primary outcome was participant interest in the recruitment message, defined as logging onto the study website by clicking the link in the message within 6 months of receiving the initial message. The secondary end point was the time-to-event outcome of clicking on the link in the recruitment message. Of note, each link contained information about which of the 4 groups the participant was randomized to so that their study group could be ascertained after landing on the study page. Recruitment messages were sent to eligible patients between September 2019 and March 2022.

### Statistical Analysis

Continuous variables were presented as medians with IQR. Categorial variables were reported in absolute numbers and percentages. Based on the prespecified analysis plan, a *P* value of .01 or less was considered statistically significant for the primary hypothesis tests to account for up to 4 interim analyses, as described later in this section. The occurrence of the primary outcome was reported as a proportion. A robust log-linear Poisson model was used to model the occurrence of the primary outcome as a function of the method of contact and content of the message. Intervention effects were reported as the relative risk (RR) of engagement along with a 99% CI. The model included the risk group phenotype as a covariate. The model was fit using generalized estimation equations to provide a robust estimate of the covariance. An independent working correlation structure for the GEE models was specified and used robust (sandwich) standard errors. The score test was used to determine whether there was a significant interaction between the recruitment message and recruitment modality. If an interaction was found, it was retained in the model, otherwise it was dropped from the model. The time-to-engagement analysis was undertaken using a Cox proportional hazard model, with covariates for recruitment message and modality. Intervention effects were reported as a hazard ratio (HR) and associated 99% CI. Exploratory subgroups of interest included older than 60 years vs 60 years or younger, sex, race, and risk group phenotype. Subgroup analyses were prespecified but exploratory, and no formal adjustment for multiplicity was applied. As a result, interaction findings should be interpreted cautiously given the increased probability of type I error. Race was self-reported by participants. In the event of no response from the participant (excluding those who did not wish to report), race was extracted from the EHR, if available. Subgroup interactions were considered to be statistically significant if *P* ≤ .05. Statistical calculations were performed using SAS version 9.4 (SAS Institute).

Up to 4 interim analyses of the study recruitment strategy were planned, and results were reviewed by an independent committee. If using a binomial test, 1 strategy was determined to be better than another, the weaker modality would be abandoned. We used an alpha spending strategy of *P* < .01, which allowed for a total of 5 comparisons, for a total family-wise error rate of .05. An important design modification occurred following the interim analysis. Prior to randomization of the third phenotype group (BMI 18-25 with 10-year ASCVD risk ≥20%), email recruitment demonstrated clear superiority over patient portal messaging. Given the limited pool of potentially eligible participants in this phenotype and the overarching priority of achieving enrollment targets for the parent RESILIENCE study, this group was subsequently recruited using email only rather than continuing randomization. Consequently, this phenotype was excluded from the present randomized comparative analysis of recruitment strategies. As a result, the analytic population for the present study represents a modified randomized cohort restricted to phenotypes that continued to undergo factorial randomization, rather than the full originally randomized sampling frame.

## Results

### Randomized Participant Demographics

Between September 2019 and March 2022, 15 376 potential participants from risk group phenotypes 1, 2, and 4 were identified using EHR data and invited to participate in the RESILIENCE study (Figure 1). Of those, 7737 (50.3%) were randomized to the email recruitment modality and 7639 (49.7%) were randomized to the patient portal modality. Of potential participants, 7682 (50.0%) received an altruistic recruitment message and 7694 (50.0%) received an individualistic recruitment message. Of all potential participants, 8240 (53.6%) were older than 60 years; 9309 (60.5%) were female; 4452 (29.0%) were Black or African American, 9346 (60.8%) were White, and 1203 (7.8%) belonged to additional groups, including American Indian or Alaska Native, Asian, Native Hawaiian or Other Pacific Islander, or more than 1 racial group; the median (IQR) BMI was 32.2 (24.2-36.4); 7557 (49.3%) were being treated for hypertension; 4853 (31.6%) had a history of diabetes; and 1307 (8.5%) were active smokers (Table 1). Baseline demographics were similar among randomized recruitment groups. With regards to phenotype, 6667 (43.4%) were in the high BMI and high ASCVD risk group (phenotype 1), 4055 (26.4%) were in the high BMI and low ASCVD risk group (phenotype 2), and 4654 (30.3%) were in the normal BMI and low ASCVD risk group (phenotype 4).

**Table 1.** Baseline Characteristics of Potential Participants Stratified by Recruitment Modality and Message

Characteristic	Individuals, No. (%)					
	All (N = 15 376)	Modality			Message	
		Email (n = 7737)		Patient portal (n = 7639)	Altruistic (n = 7682)	Individualistic (n = 7694)
Demographic						
Age, y						
>60	8240 (53.6)		4140 (53.5)	4100 (53.7)	4070 (53.0)	4170 (54.2)
≤60	7136 (46.4)		3597 (46.5)	3539 (46.3)	3612 (47.0)	3524 (45.8)
Sex						
Female	9309 (60.5)		4644 (60.0)	4665 (61.1)	4649 (60.5)	4660 (60.6)
Male	6067 (39.5)		3093 (40.0)	2974 (38.9)	3033 (39.5)	3034 (39.4)
Race^a^						
White	9346 (60.8)		4721 (61.0)	4625 (60.5)	4667 (60.8)	4679 (60.8)
Black or African American	4452 (29.0)		2221 (28.7)	2231 (29.2)	2206 (28.7)	2246 (29.2)
Additional groups^b^	1203 (7.8)		619 (8.0)	584 (7.6)	626 (8.1)	577 (7.5)
BMI, median (IQR)	32.2 (24.2-36.4)		32.2 (24.3-36.5)	32.2 (24.2-36.4)	32.2 (24.2-36.5)	32.2 (24.2-36.4)
Study phenotypes						
Obesity and high risk	6667 (43.4)		3420 (44.2)	3247 (42.5)	3329 (43.3)	3338 (43.4)
Obesity and low risk	4055 (26.4)		2008 (26.0)	2047 (26.8)	2026 (26.4)	2029 (26.4)
Nonobesity and low risk	4654 (30.3)		2309 (29.8)	2345 (30.7)	2327 (30.3)	2327 (30.2)
Comorbidities						
Hypertension	7557 (49.3)		3799 (49.2)	3758 (49.3)	3783 (49.4)	3774 (49.2)
Total cholesterol, median (IQR), mg/dL^c^	187.0 (159.0-215.0)		187.0 (159.0-215.0)	187.0 (159.0-216.0)	187.0 (160.0-215.0)	186.0 (159.0-216.0)
HDL cholesterol, median (IQR), mg/dL^d^	49.0 (40.0-61.0)		49.0 (39.0-61.0)	49.0 (40.0-62.0)	49.0 (39.0-61.0)	49.0 (40.0-62.0)
Diabetes	4853 (31.6)		2447 (31.6)	2406 (31.5)	2442 (31.8)	2411 (31.3)
Smoking	1307 (8.5)		670 (8.7)	637 (8.3)	621 (8.1)	686 (8.9)

Abbreviations: BMI, body mass index (calculated as weight in kilograms divided by height in meters squared); HDL, high-density lipoprotein.

SI conversion factor: To convert total and HDL cholesterol to millimoles per liter, multiply by 0.0259.

^a^ Race information was missing for 375 potential participants.

^b^ Additional groups included individuals identifying as American Indian or Alaska Native, Asian, more than 1 race, and Native Hawaiian or Other Pacific Islander.

^c^ Total cholesterol values missing for 2421 potential participants.

^d^ HDL cholesterol values missing for 2426 potential participants.

### Overall Response Rates by Phenotype Group and by Demographic Groups

Overall, the primary outcome (engagement as evidenced by clicking on the link in the message for the study website within 6 months) occurred in 1220 individuals (7.9%). In an exploratory subgroup analysis, the high BMI and low ASCVD risk phenotype had the highest rate of the primary outcome (408 [10.1%]). This is compared with 343 individuals (7.4%) in the normal BMI and low ASCVD risk phenotype and 469 (7.0%) in the high BMI and high ASCVD risk phenotype. No significant interaction between recruitment modality and message content was observed (*P* = .56), allowing the effects of the 2 intervention factors to be assessed separately.

### Response Rate Likelihood by Recruitment Modality

**Figure 2.**
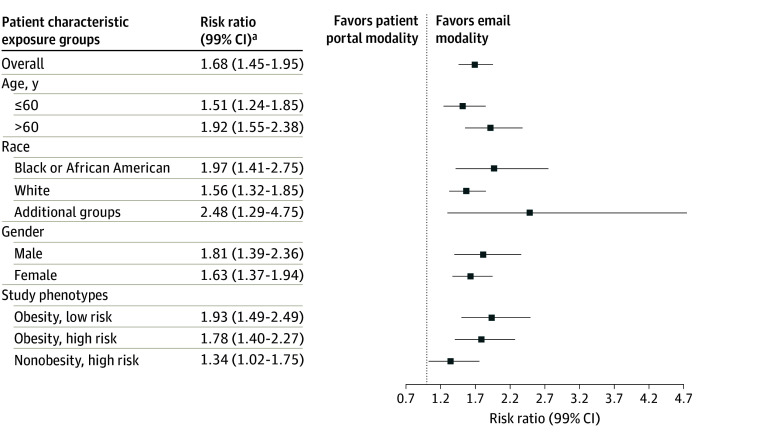
Dot Plot of Exploratory Subgroup Analysis of Recruitment Modality and Participant Engagement

The primary outcome was more likely to occur among potential participants who were randomized to receive a recruitment message by email (768 [9.9%]) than those who were randomized to receive a message through the patient portal (452 [5.9%]) (RR, 1.68; 99% CI, 1.45-1.95; *P* < .001). In an exploratory subgroup analysis, the superiority of email over patient portal message was consistent across subgroups of age, race, sex, and study phenotype (Figure 2; eTable 2 in Supplement 2). The effect was more pronounced for those older than 60 years compared with those aged 60 years or younger (>60 years: RR, 1.92; 99% CI, 1.55-2.38; ≤60 years: RR, 1.51; 99% CI, 1.24-1.85; *P* for interaction = .04). Similarly, an interaction was observed between risk phenotype and the effects of recruitment modality. The use of email was associated with greater engagement in the high BMI and low ASCVD risk phenotype (RR, 1.93; 99% CI, 1.49-2.49) compared with the high BMI and high ASCVD risk phenotype (RR, 1.78; 99% CI, 1.40-2.27) and normal BMI and low ASCVD risk phenotype (RR, 1.34; 99% CI, 1.02-1.75) (*P* for interaction = .03). While no interaction was observed between race and message modality (*P* = .06), there was a directionally greater engagement with email among participants who identified as Black or African American (RR, 1.97; 99% CI, 1.41-2.75) and additional groups (RR, 2.48; 99% CI, 1.29-4.75) compared with those who identified as White (RR, 1.56; 99% CI, 1.32-1.85). No interaction was observed between male and female individuals (male: RR, 1.81; 99% CI, 1.39-2.36; female: RR, 1.63; 99% CI, 1.37-1.94; *P* for interaction = .38). In the time-to-event analysis, email modality was associated with increased rate of message engagement (HR, 1.73; 99% CI 1.48-2.01) (Table 2).

**Table 2.** Exploratory Subgroup Analysis of Time to Engagement Outcome of Potential Participant Engagement^a^

Subgroup	Recruitment message: altruistic vs individualistic, relative risk (99% CI)	*P* for interaction	Recruitment modality: email vs patient portal, relative risk (99% CI)	*P* for interaction
Age, y				
≤60	1.23 (1.00-1.51)	.02	1.54 (1.25-1.90)	.04
>60	0.95 (0.77-1.17)		1.98 (1.58-2.47)	
Race				
White	1.09 (0.91-1.29)	.51	1.60 (1.34-1.91)	.09
Black or African American	1.02 (0.73-1.40)		2.02 (1.43-2.85)	
Additional groups^b^	1.39 (0.75-2.56)		2.53 (1.30-4.91)	
Sex				
Male	1.12 (0.86-1.45)	.74	1.85 (1.41-2.43)	.45
Female	1.07 (0.90-1.28)		1.68 (1.40-2.02)	
Study phenotypes				
Obesity and low risk	0.96 (0.75-1.24)	.20	2.02 (1.54-2.64)	.02
Obesity and high risk	1.09 (0.86-1.38)		1.82 (1.42-2.34)	
Nonobesity and low risk	1.25 (0.95-1.65)		1.35 (1.02-1.79)	

^a^ Cox proportional hazard model for time to potential participant engagement by exposure to recruitment message and modality type adjusted for study phenotype.

^b^ Additional groups included individuals identifying as American Indian or Alaska Native, Asian, more than 1 race, and Native Hawaiian or Other Pacific Islander.

### Response Rates by Recruitment Message Content

**Figure 3.**
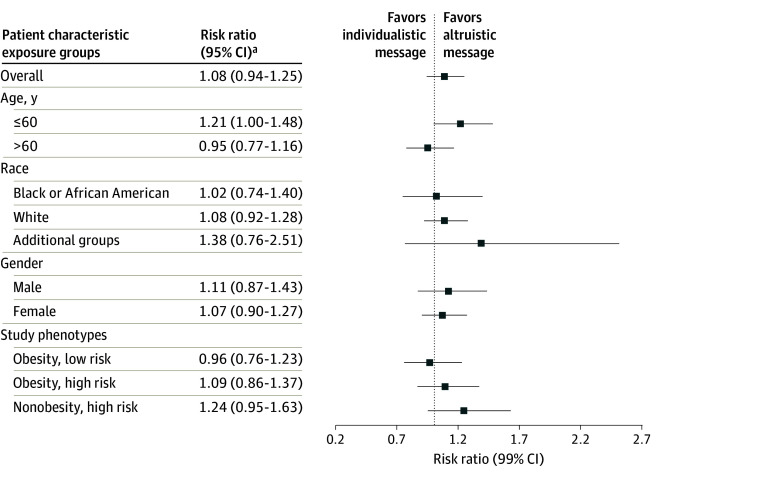
Dot Plot of Exploratory Subgroup Analysis of Message Content and Participant Engagement

No difference in the primary outcome was observed among patients who received an altruistic recruitment message (634 [8.3%]) vs an individualistic message (586 [7.6%]) (RR, 1.08; 99% CI, 0.94-1.25; *P* = .15). In the exploratory subgroup analysis, age 60 years or younger was associated with greater engagement with an altruistic message compared with those older than 60 years (≤60 years: RR, 1.21; 99% CI, 1.00-1.48; >60 years: RR, 0.95; 99% CI, 0.77-1.16; *P* for interaction = .03). No interaction between race, sex, or study phenotype was observed (Figure 3; eTable 2 in Supplement 2). In the secondary time-to-event analysis, no difference in rate of engagement was observed between altruistic vs individualistic message content (HR, 1.08; 99% CI, 0.93-1.26; *P* = .16) (Table 2)

## Discussion

While prior studies have explored the impact of various methods of contact and the content of research recruitment material, few have done so at a large scale in a randomized fashion. We found that contact via email led to a greater rate of engagement than contact through an EHR patient portal. However, the content of the message (appealing to either altruistic or individualistic values) was not associated with a difference in participant engagement. In exploratory analyses, we did observe a significant interaction regarding age, with individuals aged 60 years and younger demonstrating a signal suggesting greater responsiveness to an altruistic message compared with an individualistic message. Additionally, while all age groups were more likely to respond to an email message than a patient portal message, the magnitude of this association varied across age and study phenotype in exploratory analyses.

### Recruitment Method of Contact

While more than 90% of health systems currently use EHRs with access to a patient portal, actual portal usage is estimated to be 40% to 60% in the United States.^7,21^ In response, there has been a simultaneous growth in the use of patient portals for study recruitment.^17,22^ However, there is limited randomized data on the effectiveness of this strategy. In a randomized study of 858 healthy individuals (mean age, 38; 58% female; and 77% non-Hispanic White), patient portal recruitment rates were lower than with mailed letter and phone call strategies; however, patient portal recruitment was associated with lower costs and was preferred by participants.^14^ Similarly, in a cross-sectional study assessing adherence to public health measures related to COVID-19, recruitment rates via a patient portal were similar to postal mail but occurred faster.^23^ However, there are concerns that patient portals may lead to recruitment of less diverse populations. In a nonrandomized study examining a behavioral weight-loss intervention in adolescents and emerging adults, participant recruitment via patient portals yielded a significantly different (older, less ethnically diverse) patient population compared with clinic recruitment cohort.^24^ This can partially be attributed to differences in characteristics of individuals who use patient portals as well as the nonrandomized nature of this study.^25^ This observed difference in patient characteristics is supported by our subgroup analysis that demonstrated an interaction with age, risk factor profiles, modality of contact and engagement rate. Overall, while patient portals may represent a cost-effective method of recruitment, in our studied population email was associated with higher engagement. A likely explanation relates to differences in user experience and access pathways between the 2 modalities. Email messages are typically delivered directly to personal devices, often with notifications enabled, and can be opened with a single click. In contrast, patient portal messages usually require multiple steps, including logging into the portal, navigating to the messaging interface, and authenticating identity. These additional steps may introduce friction and reduce spontaneous engagement, particularly among individuals with lower digital literacy, less frequent portal use, or limited familiarity with health-system digital tools. Portal messages may also be perceived as administrative rather than research-related communication, further reducing responsiveness.

### Recruitment Material Content

Potentially as important as how participants are contacted is the information that this message contains and how it is presented. While recruitment material is drafted and reviewed by multiple stakeholders—study investigators, patient advisory groups, and ethics review boards—relatively little research has been undertaken to explore how this information is relayed. Various studies highlight the importance of the various aspects of recruitment material content including the length and scope of the information provided, being sent or presented by a perceived trustworthy source (primary care physician, health care practitioner, or another trial participant), and positive messaging.^26,27^ Other mechanisms that are routinely used in the creation of patient-facing materials (user testing, incorporating patient feedback, use of multimedia recruitment material) have minimal effect on recruitment.^28^ However, there may be more subtle changes to recruitment message content that center around why participating may be important to a participant.

In a clinical trial of pregnant women with gestational diabetes or impaired glucose tolerance, potential participants were randomized to receive recruitment material that contained standard information on the risk of gestational diabetes vs targeted information on gestational diabetes specific to the participants’ race and ethnicity, increasing the individualistic nature of the message. Among their 445 participants, there was no significant difference in the proportion of women who agreed to be screened or enrolled in the trial.^29^ Although that study focused on a different population, the lack of effect of one type of messaging over another is consistent with our results in this study.

Interpretation of the message framing findings from the present study should consider the limitations in construct validity. The individualistic messaging differed by phenotype to preserve contextual relevance, whereas altruistic messaging was more uniform. This heterogeneity may have diluted the intended contrast between altruistic and individualistic constructs and attenuated measurable differences in engagement. As such, the modest or null overall effect of message framing may reflect construct dilution rather than a true absence of effect. These results should not be interpreted as definitive evidence against the effectiveness of altruistic messaging, but rather as indicating that message framing effects are likely context-dependent and sensitive to how constructs are operationalized.

### Limitations

Our study has limitations that should be considered when interpreting these results. The primary outcome reflected participant interest, defined as clicking a recruitment link, and should not be interpreted as participation, enrollment, or retention. The 6-month engagement window may also capture both immediate and delayed responses, limiting interpretation as a measure of recruitment effectiveness. Generalizability is constrained by the study population and digital requirements. Participants were drawn from a single health system and required prior clinical engagement, English proficiency, internet access, email or portal availability, and digital literacy. These factors likely selected for individuals already connected to health care and technology and may limit applicability to populations with lower digital access, limited English proficiency, lower health literacy, or weaker engagement with longitudinal care. Because digital access and portal use vary by age, socioeconomic status, race and ethnicity, and rurality, recruitment effectiveness may differ in more digitally marginalized groups.

The analytic sample represents a modified randomized cohort. Following a prespecified interim analysis demonstrating superiority of email recruitment, 1 phenotype (normal BMI with high ASCVD risk) was recruited using email only and excluded from randomized comparisons. Although pragmatic, this design modification may introduce selection bias and limit generalizability to this subgroup.

Subgroup analyses were exploratory, and multiple interaction tests were conducted without adjustment for multiplicity; statistically significant findings may therefore occur by chance and should be interpreted as hypothesis-generating. Additionally, the individualistic messaging differed by phenotype to preserve contextual relevance, which may have reduced construct purity and attenuated measurable differences between messaging strategies. Finally, the study was conducted during the COVID-19 pandemic which may have affected the study through several mechanisms, such as an increased use of patient portals, an increased awareness of health status by participants, or a change of trust of health care professionals.

## Conclusions

In this randomized clinical trial of recruitment strategies, email messaging led to increased participant interest compared with patient portal messaging. While message content did not significantly impact engagement overall, exploratory analyses suggested higher engagement with altruistic messaging among younger participants. Although there is significant heterogeneity among studies that seek to identify the optimal method of contact with potential research participants and the content of research recruitment material,^28^ this is likely because willingness to participate in clinical research is heterogeneous and may be influenced by age, sex and gender, race and ethnicity, socioeconomic status, comorbidity profile and burden, and personal values and beliefs. These subgroup observations should be considered hypothesis-generating and require replication in studies specifically designed to evaluate differential recruitment effects. To increase the likelihood of adequate participant recruitment, future clinical studies should consider developing bespoke recruitment strategies that are as unique as the population they aim to recruit.

## Notes

Supplement 1.
Trial Protocol and Statistical Analysis Plan

Supplement 2. **eTable 1. **Message Content **eTable 2.** Exploratory Subgroup Analysis of Primary Outcome of Potential Participant Engagement

Supplement 3.
Data Sharing Statement
