# Supplementary material for: Messaging Modality and Content for Recruitment of Research Participants: A Randomized Clinical Trial
Source: JAMA Netw Open. 2026 May 22;9(5):e2614046. doi: 10.1001/jamanetworkopen.2026.14046 (PMC13197867; doi:10.1001/jamanetworkopen.2026.14046)
Supplement: Supplement 1. — Trial Protocol and Statistical Analysis Plan [file jamanetwopen-e2614046-s001.pdf]

Study Protocol Template
Protocol Version: 1.0

Study Name: RESILIENCE:Research for pErSonalized cardIovascuLar dIsease prEvention, treatmeNt, and CarE.
Study Sponsor: Duke University – Translating Duke Health Initiative
Protocol Author: Neha Pagidipati, MD MPH

Protocol Synopsis:

- **Background/Rationale**: Obesity is a rapidly growing epidemic that is associated with the development of cardiovascular disease (CVD). However, obesity is a heterogeneous disease; while some develop CVD early, others appear to be CVD resistant. Similarly, responses to weight loss interventions in obese individual are also quite variable. While some lose weight and improve CVD risk factors, demonstrating apparent resilience to obesity and CVD risk, others are non-responsive to interventions.
- **Objectives/Hypotheses**: Our overall objective is to understand factors contributing to the heterogeneity of CVD resistance and resilience among individuals with obesity at Duke. A secondary objective is to determine optimal methods of recruiting and consenting patients for clinical studies.
- **Study Design**: The RESILIENCE study is a prospective study that will be conducted with 600 patients within the Duke Health System. We aim to recruit patients with 4 phenotypes: 200 patients with obesity (BMI ≥ 30) and high 10-year ASCVD risk (≥20%), 200 patients with obesity (BMI ≥ 30) and low 10-year ASCVD risk (<7.5%), 100 patients with normal weight (BMI 18-25) and high 10-year ASCVD risk (≥20%), and 100 patients with normal weight (BMI 18-25) and low 10-year ASCVD risk (<7.5%). Clinical, behavioral, and molecular characteristics will be compared at baseline between the 4 groups to understand heterogeneity between obesity and risk for CVD. Patients with obesity will undergo a 6-month weight loss intervention. Clinical, behavioral, and molecular characteristics will be compared between baseline and 6 months to understand (a) predictors of response to the intervention and (b) how these factors change with weight loss. To address the secondary objective listed above, we will test recruitment strategies using a 2x2 factorial design that will randomize individuals, stratified by phenotype above, to personal email vs. MyChart message, and to different message content. We will also test consenting strategies by randomizing individuals, again stratified by phenotype above, to 1 of 4 informed consent formats: text only, text + physician video, text + patient video, text + animated video.
- **Study Population**: The study population will consist of adult patients who receive care in the Duke Health System, and who fall into 1 of 4 phenotypes: 200 patients with obesity (BMI ≥ 30) and high 10-year ASCVD risk (≥20%), 200 patients with obesity (BMI ≥ 30) and low 10-year ASCVD risk (<7.5%), 100 patients with normal weight (BMI 18-25) and high 10-year ASCVD risk (≥20%), and 100 patients with normal weight (BMI 18-25) and low 10-year ASCVD risk (<7.5%). Potentially eligible patients will be identified using Duke Electronic Health Record (EHR) data, with additional inclusion and exclusion criteria.
- **Exposure(s)**: Individuals with obesity will undergo a 6-month weight loss intervention called TRACK, which is a digital behavioral intervention involving text messaging, phone-based coaching, and digital scales; it has been proven to lead to significant weight loss.
- **Outcome(s):** Differences in branched-chain amino acids will be compared between all groups, both at baseline and at 6 months (for those patients undergoing the TRACK intervention). Other clinical, behavioral, metabolomic, genetic, and microbiome parameters will also be compared in an exploratory fashion.

Protocol:

1. Background and Rationale

1.1 Background

Obesity is a rapidly growing epidemic. The global prevalence of obesity is 13%,[1] and more than a third of Americans are obese.[2] Obesity is even more widespread in our local community. Over 40% of Duke patients are obese, and only a small number of them lose weight when followed longitudinally. Obesity poses a substantial threat to patient health, and is strongly associated with cardiovascular disease (CVD) risk factors (hypertension, diabetes, hyperlipidemia) as well as to CVD itself.[3-5] Clearly, effective strategies to address the obesity epidemic on a population scale are needed to stem the growing burden of CVD. However, obesity is a heterogeneous disease; while some develop CVD early, others appear to be CVD resistant. This heterogeneity is reflected by the range of cardiometabolic abnormalities and CV events experienced by individuals with obesity.[6,7] Similarly, responses to weight loss interventions in obese individual are also quite variable. While some lose weight and alleviate CVD risk factors, demonstrating apparent resilience to obesity and CVD risk, others do not respond to interventions. Thus, there is a clinical need to understand the mechanisms of successful weight reduction and CVD risk factor improvement in patients with obesity.

1.2 Rationale

Understanding heterogeneity in weight loss and accompanying changes in CVD risk profiles at both epidemiological and biological levels is critical to providing personalized obesity care and to promoting CV health. In order to develop a complete picture of CVD resistance and resilience in obesity, we need to integrate all potential predictors to create a detailed map of individual clinical, behavioral, and molecular changes that occur in obesity. [8,9] As recently advocated for by the National Institutes of Health in *JAMA*, this requires phenotyping large numbers of patients clinically, behaviorally, and physiologically, and integrating all of these data.[10] Unfortunately, very few such data sources currently exist.

To accomplish this goal, we will identify a heterogeneous cohort of obese and normal BMI patients with high or low 10-year risk for CVD (i.e. >30% or <10%). We will then remotely collect data using electronic consents, electronic health record (EHR) clinical data, and online behavioral surveys. Further, we will collect biological data remotely with home biospecimen kits. Remote collection will reduce participant burden, increase participation and retention, and potentially reduce costs.

This platform will enable us to understand which clinical, behavioral, and biological markers are related to high-risk obese individuals vs. “resistant” low-risk obese and high-risk normal BMI individuals. Then, we will embed within this cohort a digital health behavioral intervention called TRACK. This will enable us to identify which clinical, behavioral, and biological markers change with weight loss, as well as which markers predict degree of weight loss and improvement in cardiometabolic risk factors (“resilience”).

One promising marker that we will specifically evaluate is branched chain amino acids (BCAA), which have previously been associated with insulin resistance in obese patients.[11] We will assess whether BCAA are associated with CVD risk more strongly in obese compared with normal BMI patients, and whether BCAA predict response to a weight loss intervention. We will also perform metabolomic, genetic, and microbiome profiling for discovery science.

2. Objectives and Hypotheses

Our primary objective is to understand a broad range of factors that contribute to the heterogeneity of CVD resistance and resilience among individuals with obesity at Duke. A secondary objective is to determine optimal methods of recruiting and consenting patients for clinical studies.

2.1. Primary Aims & Hypotheses

Aim 1: CVD Resistance in Obesity. We aim to understand the clinical, behavioral, and biological factors associated with obesity and CVD risk by understanding differences between obese vs. normal BMI and high vs. low CVD risk groups. To do this, we will develop a cohort of obese and normal BMI patients at high and low CVD risk (n=600), and will deeply phenotype these individuals with behavioral data and biospecimen collection. Half (n=300) will also undergo CT testing to determine coronary artery calcium (CAC) score, for validation of the above associations. Finally, an embedded mechanistic study (n= 40) will explore the effect of the identified markers on vascular endothelial function.
Hypothesis 1a: BCAA are associated with CVD risk in obese individuals more than in normal BMI individuals in a real-world population, and this association will be validated with CAC score.
Hypothesis 1b: Specific clinical, behavioral, metabolomic, genetic, and microbiome profiles will be associated with CVD risk in obese individuals more than in normal BMI individuals.
Hypothesis 1c: In tissue-engineered blood vessels, BCAA interact with lipoproteins to induce endothelial dysfunction and increased monocyte/macrophage accumulation in the arterial wall, which are early markers of atherosclerosis.

Aim 2: CVD and Obesity Resilience. We will implement a proven, digital health behavioral intervention with 400 deeply phenotyped obese individuals in our cohort. We will then explore clinical, behavioral, and biological factors associated with heterogeneity in response to this intervention. Response will be defined as (1) weight loss and (2) changes in CVD risk factors, including LDL-cholesterol, HbA1c, Homeostasis model assessment for insulin resistance (HOMA), and hs-CRP.
Hypothesis 2a: Baseline clinical, behavioral, and/or biological markers, and specifically BCAA, will predict response to the intervention.
Hypothesis 2b: Biological markers, including BCAA and other markers discovered through integrated -omics analysis, will change from baseline with weight loss and changes in CVD risk factors.
Hypothesis 2c: Obese individuals with high CVD risk who lose weight with the intervention will develop biological profiles more similar to normal BMI, low CVD risk individuals.

2.2. Secondary Aims & Hypotheses

Aim 3: Optimal Recruitment Strategies. We aim to determine optimal strategies to recruit patients into the study remotely, i.e. without requiring a formal clinic visit or the direct involvement of the patient's clinician. Specifically, we want to understand whether an initial message through personal email vs. MyChart message leads to greater engagement, and whether the content of the message affects engagement. We will do this by randomizing potentially eligible patients in a 2x2 factorial fashion to personal email vs. MyChart message, and to different message content.
Hypothesis 3a: An introductory message delivered through MyChart will lead to greater engagement (as assessed by clicking on the study website page to get more information) than will a message delivered through personal email.
Hypothesis 3b: For patients with obesity, a message conveying altruism will lead to greater engagement than a message conveying personal benefit.
Hypothesis 3c: For patients without obesity, a message conveying altruism will lead to greater engagement than a message conveying scientific importance.

Aim 4: Optimal Consenting Strategies. We aim to determine optimal strategies to consent patients into the study remotely, i.e. without requiring a formal clinic visit or the direct involvement of the patient's clinician. Specifically, we want to understand which of the following formats leads to greater completion of the consent form and greater participant comprehension of the consent form: text only vs. text + physician video vs. text + patient video vs. text + animated video.
Hypothesis 4a: An informed consent form that includes both text and an animated video will lead to greater completion of the consent form and greater comprehension of the consent form compared with a text only, physician video, or patient video consent form.

3. Study Design

3.1 Overall Study Design

The RESILIENCE study is a prospective study that will be conducted with 600 patients within the Duke Health System. We aim to recruit patients with 4 phenotypes: 200 patients with obesity (BMI ≥ 30) and high 10-year ASCVD risk (≥20%), 200 patients with obesity (BMI ≥ 30) and low 10-year ASCVD risk (<7.5%), 100 patients with normal weight (BMI 18-25) and high 10-year ASCVD risk (≥20%), and 100 patients with normal weight (BMI 18-25) and low 10-year ASCVD risk (<7.5%). We will test recruitment strategies using a 2x2 factorial design that will randomize individuals, stratified by phenotype above, to personal email vs. MyChart message, and to different message content. We will also test consenting strategies by randomizing individuals, again stratified by phenotype above, to 1 of 4 informed consent formats: text only, text + physician video, text + patient video, and text + animated video.

After consent and enrollment, baseline clinical data from the patients' electronic health record will be collected in a database. All patients will be asked to complete a series of behavioral surveys online. They will also be sent a home biospecimen collection kit, with which they will collect and return saliva and stool samples. Once the specimens have been returned, all patients will come for a visit to the Duke South Durham Clinic to have a BMI check and to have fasting labs drawn. At that point, patients with normal weight will exit the study, and patients with obesity will enter into a 6 month weight loss intervention. The intervention, called TRACK, is a proven weight loss program that utilizes health coaching and goal-setting to help patients lose weight. It is a remote intervention that is delivered entirely over their phones. The intervention will be introduced to the participants at the clinic visit, and they will receive a Fitbit and an electronic scale. At the end of the 6 month intervention, the 400 patients will be asked to collect stool specimens again with a home biospecimen kit, fill out additional behavioral surveys online, and have fasting labs drawn.

Clinical, behavioral, and molecular characteristics will be compared at baseline between the 4 groups to address Aim 1. Among patients with obesity, clinical, behavioral, and molecular characteristics will be compared between baseline and 6 months to understand (a) predictors of response to the intervention and (b) how these factors change with weight loss (Aim 2).

3.2 Study Population

The study population will consist of adult patients who receive care in the Duke Health System, and who fall into 1 of 4 phenotypes: 200 patients with obesity (BMI ≥ 30) and high 10-year ASCVD risk (≥20%), 200 patients with obesity (BMI ≥ 30) and low 10-year ASCVD risk (<7.5%), 100 patients with normal weight (BMI 18-25) and high 10-year ASCVD risk (≥20%), and 100 patients with normal weight (BMI 18-25) and low 10-year ASCVD risk (<7.5%). Potentially eligible patients will be identified using Duke EHR data, with the inclusion and exclusion criteria outlined below (see IRB protocol PRO00103647). ASCVD risk over 10 years will be determined using the Pooled Cohorts Equation.[12] For blood pressure and cholesterol (Total Cholesterol & HDL) measures, we will calculate the empirical 1% and 99%

quantiles. Any measures more extreme than these values will be truncated to the 1% and 99% respectively. For those missing any measurements (smoking, cholesterol, etc.), the worst and best case scenarios (based on the 1% and 99%) will be imputed. If a participant's ASCVD risk score is still low risk under the worst case scenario or high risk under the best case scenario, they will be categorized as such. Otherwise they will be excluded from analysis.

Inclusion Criteria:
- Adults 40-75 years old
- At least one clinic encounter at Duke with BMI record in the EHR within previous year
- Has a current primary care provider listed in EHR
- No prior history of ASCVD, as defined by ICD9, ICD10, and CPT codes for coronary artery disease, myocardial infarction, stroke, peripheral arterial disease, prior revascularization for coronary, cerebral, or peripheral arteries.
- Fall into 1 of 4 categories: 200 patients with obesity (BMI ≥ 30) and high 10-year ASCVD risk (≥20%), 200 patients with obesity (BMI ≥ 30) and low 10-year ASCVD risk (<7.5%), 100 patients with normal weight (BMI 18-25) and high 10-year ASCVD risk (≥20%), and 100 patients with normal weight (BMI 18-25) and low 10-year ASCVD risk (<7.5%).
- Have internet access
- Have an email address listed in the EHR
- Have access to MyChart
- Have a smartphone
- Be able to read and understand English

Exclusion Criteria:
- Patient "opted out" of being contacted for research in Maestro Care
- Pregnant at the time of enrollment or <12 months post-partum
- Prior bariatric surgery

4. Study Schedule

4.1 Recruitment

Recruitment will be performed via Electronic recruitment or 'eRecruitment'. It will involve identification of potentially eligible participants within the Duke Electronic Health Record and reaching out them via electronic means.

4.1.1 Electronic Recruitment: 'eRecruitment'
Once identified as potentially eligible for the study, individuals will be assigned a recruitment/consent strategy based on block randomization of the 4 phenotypes (see Section 5.1). Two recruitment modalities and two recruitment messages will be tested in a 2x2 fashion (see Figure 1, below.).

Figure 1. 2 x 2 factorial randomization of method of contact and content of messages.

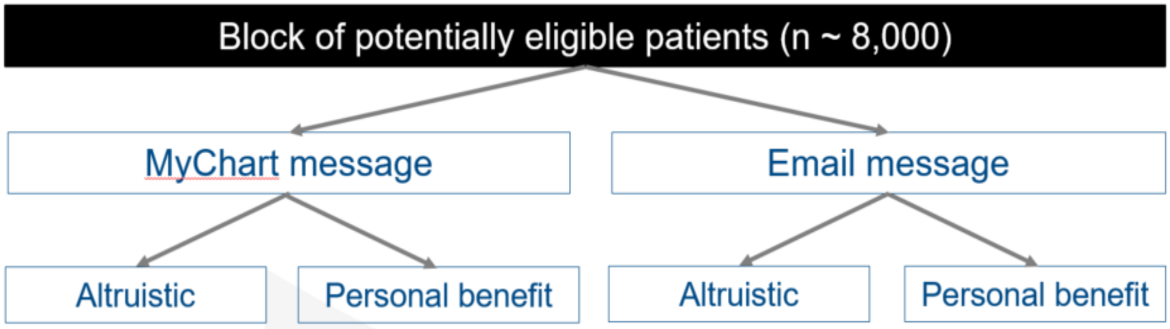

4.1.1.1 Method of contact

While direct to patient engagement has been touted as an important method for obtaining a broad cohort of patients, little is known about which medium is most effective at recruitment. Here we intend to compare the use of a generic 'hospital' email with the use of the Duke 'MyChart' message service. From a cohort identified within the Duke EMR as being potentially eligible, potential participants will be randomized to receive either an email or a message through their Duke MyChart. Emails will be sent from a secure server within Duke University Technology Services. The MyChart message will be disseminated through Maestro Care under the guidance of the Duke Office for Clinical Research.

4.1.1.2 Content of messages

Recruitment of patients in clinical research is low and it remains unclear what aspects of the research enterprise motivate their involvement. We aim to explore whether the content of our initial messaging has varying impact on initial engagement in the study. Specifically, we are interested whether an altruistic purpose (e.g., the accrual of scientific benefit for others) compared to a more egocentric purpose (e.g., the hope of improving one's own health) is more motivational for participation (see content below Table 1).

Table 1. Content of messages

Obese group		Lean group	
**Altruistic**	**Personal Benefit**	**Altruistic**	**Science-centric**
Every day millions of people in the US struggle with their weight. Some respond to weight loss treatments while others do not. Likewise, some develop heart disease while others do not. We are doing the RESILIENCE study to understand more about these differences so that we can develop better treatments.<br><br>You may be a good fit for this study. Please think about joining Duke researchers in a study that will lead to better treatments for millions of people living with	The RESILIENCE study aims to help people who want to lose weight and improve their heart health. We want to understand why some people with weight issues develop heart disease while others do not. Likewise, we want to understand why some people respond to weight loss treatments while others do not.<br><br>Participating in this study may make it possible for you to get better weight management and heart health treatments in the future. Please consider joining us in the RESILIENCE study!	Millions of people in the US are at risk for heart disease, and weight is an important part of this risk. We are doing the RESLIENCE Study to understand the differences in heart health between people who have obesity and those who do not.<br><br>Although you are not overweight, by joining this study you can help us understand these differences.<br><br>Please consider partnering with Duke researchers on a study that will lead to improved heart health for many people in the future.	We know that weight is an important part of heart health. However, you may be surprised to know that we do not yet fully understand how weight affects heart health.<br><br>We are doing the RESILIENCE study to understand why some people with obesity develop heart disease while others do not. Although you are not overweight, by joining the study, you can help us discover an answer to this important scientific question.<br><br>Please consider joining

obesity.			Duke researchers in the RESILIENCE study, and advancing science to prevent heart disease.

4.1.1.3 Assessment of engagement

All individuals will be randomized upfront as MyChart vs. e-mail invites. Obese group message will further be randomized as altruistic vs. personal benefit and non-obese group as altruistic vs. science focused. They all will also be provided with a unique 'tracking' URL which in turn will utilize application of FHIR ID (a unique identifying number that links specific participant information to Mytonomy). In order to determine the comparative effectiveness of the message content, data will be collected on the number of people who click on the 'I'm interested' button and subsequently arrive at the 'landing' page for further information. The arrival at the 'landing' page (which contains further information about the trial and further links to the eConsent portal) will be tracked using the original URL and embedded FHIR ID which after decryption will allow association back to identifying them as Duke patient with their first and last name, email address and mobile phone number. This will facilitate not only an understanding of which method is overall most successful in engaging patients (in aggregate) but also allow us to understand what type of approach is more likely to recruit certain types of patients (e.g. age, sex, ethnicity, geography etc.).

4.1.2.1 Confirmation of eligibility

Participants will be identified ahead of time based on inclusion/exclusion criteria from their Duke-EMR records. Eligibility will further be confirmed upon their arrival at the clinic for baseline visit.

4.1.2.2 Assessment of engagement

Participants recruited via eRecruitment pathway will be assessed to understand their level of engagement of study participation (e.g. participants viewing the MyChart vs. email messages, number of reminder attempts to consent from landing page, those who consent vs don't consent etc.).

4.2 Consent and Enrollment

Low rates of patient participation in clinical research jeopardize the generalizability of research findings and represents a disparity in access to research and subsequent clinical care. Pragmatic research methods have ameliorated some of the trial-related burden for patients, however there exists a number of barriers to enrollment. One significant obstacle is informed consent. Although conceived to educate on the rationale, risks and benefits of clinical research, a focus on legal disclosure has resulted in the consent process becoming lengthy (frequently >15 pages) and complex (greater than grade 12th literacy), and may represent a key deterrent to participation. This barrier is amplified in individuals with reduced literacy skills and may explain some of the disproportionate enrolment within key groups such as non-white race, non-English speaking and the elderly. Furthermore some patients may have historically participated without adequate understanding, thereby consenting in an 'uninformed' manner.

Video-based consent has been raised as a potential method to improve the comprehension of the consent process and mitigate enrolment barriers. A video-based consent method leverages conversational language, augments understanding through graphics and animation, and facilitates consent at their own pace. In a proof of concept study by our group published in Circulation: Cardiovascular Quality and Outcomes, video-based consent was associated with greater recruitment metrics and increased enrolment of non-white and elderly patients (Fanaroff AC et al. Circ Cardiovasc Qual Outcomes 2018 Apr;11(4):e004675).

In this study we will not only be testing the utility of video-based compared to 'text based' consent in a randomized manner, but also whether there are differences in the performance of video-based consent depending on who provides the information.

4.2.1 eConsent portal

Once potential participants arrive at the study landing page hosted by Mytonomy, Inc. through the eRecruitment process), they will see a brief description of the study along with an introductory video. Individuals will then be able to click on a 'Click to consent' link to begin the consent process. All of participants' responses and profile information will be kept confidential in a HIPAA-compliant Data Server hosted in Amazon Web Services by Mytonomy (data server information provided by Mytonomy).

4.2.2 eConsent randomization

Consecutive individuals who click on the 'click to consent' on the 'landing' page will be randomized to consent via one of four methods: a) video consent with study information provided by patient, b) video consent with study information provided by doctor, c) video consent with study information provided by animation/cartoon or d) text-based study information and consent (standard of care).

4.2.3 eConsent sub-study

Potential participants will be presented with the first page of the consent portal which will provide them IRB-approved consent form to review to sign and view one of the 4-types of consent-content mentioned above that they will be randomized to. Consent can be an important barrier to recruiting a broad cohort of patients and we are striving to include everyone in this type of research. We would like to collect some basic information to understand what types of consent work for different people. In order to do so, we'd collect consent-content viewership information, numbers enrolled against type of consent video/text presented and ICF-comprehension questionnaire. .

4.2.4 eConsent tracking

Once enrollment is complete, the study team will be able to evaluate the number of potential participants who started vs. completed the consent form. This will not only provide us with information about the overall 'most' successful consent medium but also the proportion of patients who started and did not finish within each consent 'arm'. Moreover, from their basic demographic information we will be able to understand what types of people do not proceed through varying consent methods.

4.2.5 eConsent arms

Each of the three videos will follow the exact same content script however will differ in their delivery. One video will involve a patient (or actor portraying a patient) talking about the study and walking the potential participant through the study consent. The other videos will use a doctor or an animated figure to do the same. If the participant is randomized to the text consent, the informed consent form material will simply be provided in text format.

4.2.6 eConsent comprehension

Through the use of conversational verbiage and the ability to stop, start and rewind, our group believes the eConsent will result in greater participant comprehension of the study – thus, a more *informed* consent. In turn this may lead to a more engaged trial participant who is more likely to complete the study and not be lost to follow up. This has not been shown in a randomized manner and thus we will be evaluating comprehension across all four arms of the consent: three videos and one text-based.

Upon signing the form each participant will be asked a total of five questions. If the answer is incorrect the participant will be provided with the correct answer, but this will not prevent them from signing the

consent form. At the completion of this <2-minute quiz, participants will be able to electronically sign the form and have a PDF of the signed form emailed to their personal email address.

Figure 2. Consent pathway.

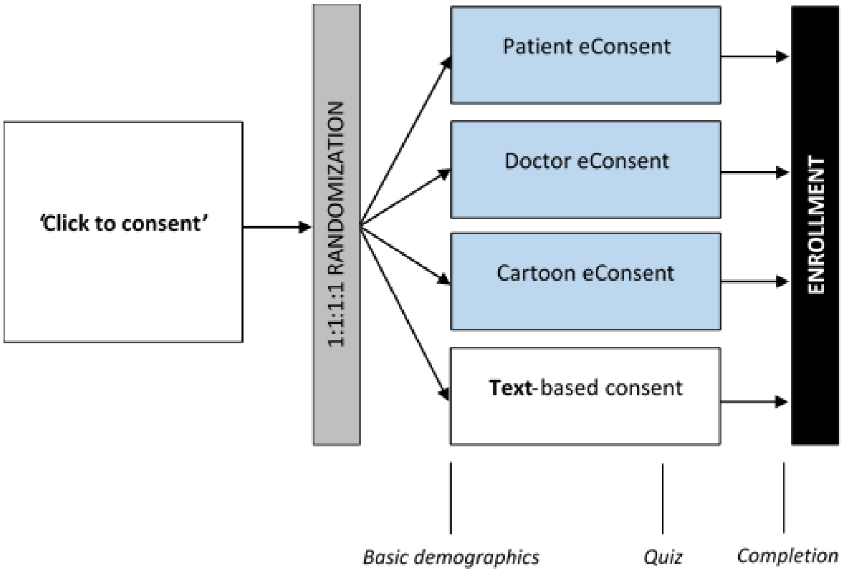

4.3 Baseline Assessments

Once consented, participants in all 4 groups will be asked to complete two tasks at home (behavioral surveys and home biospecimen kits); directions for these tasks will be delivered to them via Mytonomy's online platform.

First, participants will complete a series of online behavioral surveys available via Mytonomy's platform. These surveys will take approximately 15-20 minutes to complete and can be completed over multiple sessions.

Second, a biospecimen collection kit will be sent to each participant's home. As a patient is enrolled in the study, a kit label will be generated using participants' home address. A study team member will affix the label and scan it to PEDIGENE®. The kit will then be mailed to each participant's home via the US Postal Service and tracked via USPS tracking system.

Each kit will contain 3 items: (1) instruction sheet, (2) Oragene saliva collection kit, and (3) stool specimen collection kit. The instruction sheet will outline how to collect, package, and store each specimen; it will also contain a link to an online video that will walk participants through the biospecimen collection process.

Patients will have approximately 2 weeks to return their samples. The 3 specimen collection items will be mailed back via the US Postal Service using pre-fixed labels.

If participants do not return the saliva and stool kits to the study team within 3 weeks, they will be called as a reminder, and the CRC will address questions/concerns if necessary. If they still have not returned

the kits within 5 weeks, they will receive an email to let them know that they will not be able to continue in the study.

Those who return the saliva and stool samples to the study team will proceed to the next part of the baseline assessments. The CRC will call the participant to schedule an in-person visit and fasting lab draw at the Duke South Durham Clinic at 234 Crooked Creek Pkwy, Durham NC, 27713. At the visit, all participants will meet with the CRC to have any questions answered, complete behavioral surveys on iPad if not already done, measure height and weight on a calibrated weighing scale, have fasting labs drawn (lipid panel, HbA1c, glucose, insulin level, hs-CRP and metabolomic analyses (research draw)) and receive compensation via ClinCard. In addition, the 300 study participants who will receive a CAC score will be consented for this procedure in-person. Their CAC test will be scheduled at this time. After that, participants without obesity will exit the study.

4.4 Weight Loss Intervention

Individuals with obesity will continue in the study. At the same clinic visit, in addition to the procedures outlined above, individuals with obesity will be introduced to the TRACK weight loss intervention. Enrollment information, including name, phone number and email address, will be entered into the TRACK platform for participant registration. Participants will then receive a cellular connected scale and Fitbit, directions on downloading and linking their Fitbit, and an orientation to the intervention.

After the study visit, participants will enter a 2-week period to get baseline physical activity data on their Fitbit device before starting the TRACK intervention. After 2 weeks, the TRACK intervention will begin.

Table 2. Intervention design

Component	Frequency	Target
**Telephone counseling calls from dietitian**	Calls 1-4: weekly<br>Calls 5-10: bi-weekly<br>Calls 11-12: monthly	Social persuasion<br>Skills training<br>Social support
**Obesogenic behavior change goals**	Daily	Goal setting
**Self-monitoring with tailored feedback**	$\geq$ 1x/wk	Mastery experiences<br>Somatic/emotional reactions
**Tailored skills training materials**	Sent quarterly	Mastery experiences<br>Social modeling<br>Skills training

<u>Obesogenic behavior change goals</u>: Participants will track 4 behavior change goals that directly/indirectly produce an energy deficit. As part of the baseline behavioral surveys, each participant will have completed a short, self-administered survey to assess level of engagement in various dietary, physical activity, and other weight control behaviors. A computer algorithm then uses this information to create a personalized ranking of all the goals in the library based on each participant's need to change each behavior, readiness and self-efficacy to change each behavior, and the potential caloric deficit promoted by the specific behavior change. The algorithm rank orders the goals and participants are asked to self-monitor their adherence to the top 3 goals for the first 8-weeks of the study. Then, starting at week 9, participants self-monitor the next 3 goals on their list in order to maintain motivation and facilitate goal mastery. Goals change every 8 weeks throughout the 6-month intervention period. All participants also receive a universal 4th goal that rotates at 8-week intervals. In the first interval, we assign a “no red zone foods” goal. To determine the “red zone foods,” we ask participants to select the foods they consume regularly (at least 3 days per week) from a list of commonly eaten, high-calorie foods and beverages (e.g., sodas, sweet tea, desserts, potato chips, pizza, hamburgers). This goal encourages participants to reduce the highest-calorie foods in their diet and maximize the caloric deficit. We provide all intervention participants with a list of “green zone” foods that they can substitute for their red zone foods. The other universal goals are: “practice portion control” and “walk 7–10,000 steps per day.”

Our goal library includes the following: 1) no sugar-sweetened beverages; 2) no fast food; 3) no late night snacking; 4) eating breakfast daily; 5) eating 5 to 9 servings of fruits and vegetables daily; 6) achieving 10,000 steps per day, in increments of 2,000 and 3,000 steps/day over baseline; 7) no consumption of high calorie snacks; 8) no more than 2 hours of TV daily; 9) having low-fat dairy instead of high-fat dairy; 10) no fried food; 11) eating red meat once per week or less; 12) doing brisk activity 30 minutes 5 times per week; 13) no more than 1 alcoholic drink per day; 14) going to the YMCA 3 times per week; 15) daily self-weighing; 16) 7-8 hours of sleep each night; 17) replacing white grains with whole wheat; 18) no seconds at meals; 19) using a smaller plate and fork at meals; 20) strength training 2-3 times per week; and 21) no high-fat seasonings.

<u>Self-monitoring</u>: Regular self-monitoring is a robust predictor of weight loss, although adherence wanes over time. To enhance engagement potential, intervention participants will choose to self-monitor via interactive voice response (IVR) or SMS text messaging. Tailored self-monitoring feedback is immediately provided through both IVR and text. Feedback messages describe trends in participant progress, reinforce successes and/or offer motivational strategies. Finally, a short skills training message is provided. Each week, we will also provide an engaging summary feedback message, patterned on a strategy used in Shape Plan.

Regular self-monitoring of body weight is supported by emerging evidence. As in previous studies, we will provide intervention participants with scales from BodyTrace, which transmit weight data directly to our systems through the cellular network; they do not need a computer or Internet connection. Thus, weight data is objective and can be used in counseling and feedback. We will ask participants to weigh themselves daily and we will provide materials to help participants interpret their daily weight fluctuations.

<u>Tailored skills training materials</u>: Tailored strategies can heighten the personal relevance of content, while minimizing cost and labor intensity. Our materials are tailored at several levels. Participants will receive skills training content that corresponds to their behavior change goals. Skills training content will be tailored based on gender, language, participant motivation, and parental status. We will adapt our extensive library of existing materials, which includes information about cost and community resources. At the baseline visit, participants will receive a set of tailored intervention materials to be utilized over the first 3 months of the intervention. Study staff will send participants (via mail, email, web, text) additional materials every 3 months with goal changes. We will conduct literacy assessments on all materials.

<u>Telephone counseling calls</u>: Health coaches will deliver counseling calls weekly in weeks 1-4, biweekly in months 2-4 and monthly calls in months 5-6, for a total of 12 calls over the 6-month intervention period. Each 20-30 minute counseling call will enhance/sustain participant motivation, deliver in-depth behavioral skills training, and provide social support. Counseling will be guided by principles of motivational interviewing (MI). MI enhances self-efficacy, increases recognition of inconsistencies between actual and recommended behaviors, and teaches dissonance reduction skills. On each call, interventionists will: (1) review self-monitoring data and reinforce its importance; (2) discuss barrier reduction strategies; (3) deliver skills training content, and; (4) discuss engaging community resources. In later sessions, interventionists and patients will collaboratively develop weight maintenance plans.

Interventionists will use a web application that presents each session’s call script, allows for note taking, and provides access to self-monitoring data. The system can record calls and automatically stores process data (e.g. date/time, call disposition, duration). We will train interventionists using protocols proven effective in our prior trials.

4.5 End of Study Assessments

At the end of the 6 month intervention, each of the 400 participants will again be given a series of online behavioral surveys that will be administered through the Mytonomy platform. These surveys will take approximately 15-20 minutes to complete in total, and can be completed over multiple sessions.

In addition, participants will be mailed another biospecimen collection kit. The contents and procedures will be the same as at baseline, EXCEPT saliva will not be collected again; only stool collection kits will be included. The samples will be tracked, mailed, and returned in the same way as at baseline. Participants will have approximately 2 weeks to return the kits; after that, they will be called by the CRC as a reminder.

Once the samples have been returned, the CRC will call each participant to schedule a fasting lab draw at the Duke South Durham Clinic at 234 Crooked Creek Pkwy, Durham NC, 27713. After that point, the participants will exit the study.

4.6 Schedule of Assessments

		Baseline (Non-obese Participants)	Baseline (Obese Participants)	6-months intervention completion (Obese Participants)
Remote Assessments				
	E-informed consent	X	X	
	Oragene saliva collection	X	X	
	Stool specimen collection	X	X	X
	Behavioral surveys	X	X	X
Clinic Visit Assessments				
	Informed consent for Basic Science sub-study (30 participants)	X	X	
	Informed consent for CT testing for CAC score (300 participants)	X	X	
	Height	X	X	
	Body weight	X	X	X
	Lipid panel	X	X	X
	HbA1c	X	X	X
	Glucose	X	X	X
	Insulin level	X	X	X
	hs-CRP	X	X	X
	Research blood draw[a]	X	X	X
	Basic Science sub-study blood draw[b]	X	X	X
	Coronary calcium CT[c]	X	X	

Abbreviations: HbA1c, hemoglobin A1c; hs-CRP, high-sensitivity C-reactive protein

[a] This research blood draw is for metabolomics and genetic analyses and biorepository storage

[b] 50 mL of whole blood per individual will be collected per visit from 30 participants in the following groups: 15-obese high-risk (before and after intervention), 15-obese low-risk (before and after intervention)

[c] 300 participants will perform CT testing for CAC score: 150 non-obese participants and 150 obese participants

4.7 Basic Science Sub-study

Purpose and objective: In the basic science sub-study, blood will be drawn for the isolation of endothelial colony forming cells to examine the effect of altered plasma branched chain amino acid (BCAA) levels on vascular function.

Study activities and population group: We will draw 50 mL of whole blood per visit from individuals in the following groups:

- 15 obese high-risk subjects, before and after intervention
- 15 obese low-risk subjects, before and after intervention

Participants will be approached on a first come first serve basis until enrollment for the sub-study is met. The blood will be processed to isolate the endothelial colony forming cells.

Data Analysis and risk/safety issues: The risks of a venous blood draw in a healthy adult volunteer are minimal and includes pain, some redness, and a very low risk of infection in the area. Once the blood is processed, and the cells are isolated and studied, we will fabricate tissue-engineered blood vessels to evaluate a patient-specific response to different levels of BCAA.

Primary Objective: Our goal is to establish whether chronic exposure to altered BCAA levels in blood plasma alters vascular function. Specifically, we will test the hypothesis that in tissue-engineered blood vessels, BCAA interact with lipoproteins to induce early markers of atherosclerosis, such as endothelial dysfunction and increased monocyte/macrophage accumulation in the arterial wall.

Overall study plan: We hypothesize that the elevation of BCAA in obesity and cardiovascular disease, together with altered levels of plasma lipids (in the form of low density lipoproteins (LDL)), increase endothelial dysfunction and monocyte accumulation in the blood vessel wall. To test this hypothesis, we will use cocultures of endothelial cells (ECs) and smooth muscle cells (SMCs) within tissue-engineered blood vessels (TEBVs). First, we will examine interactions of BCAA, with and without elevated modified LDL, on endothelial responses to flow. We will expose endothelial cells grown in flow chambers to different concentration profiles of BCAA found in obese individuals at low- or high-risk for cardiovascular disease, with and without modified LDL. In these endothelial cell cultures, we will examine inflammation, the generation of reactive oxygen species, and the activity of the proteins eNOS and mTOR. We will examine different combinations of BCAA concentrations to identify which has the most significant effect on disease-mediating proinflammatory and prooxidant gene expression. Next, for those combinations of BCAA found to induce the most significant pathological effects on endothelial cells, we will examine their impact on TEBV function. We will perfuse TEBVs with the most pro-atherogenic conditions identified in the previous endothelial cell experiments. These TEBVs will be made with human cells and perfused for 1-6 weeks in order to examine the hallmark signs of early atherosclerosis: decreased vasoactivity, increased adhesion of circulating monocytes, and foam cell formation from adherent monocytes and smooth muscle cells. We expect to observe a relationship between BCAA combinations present in high-risk individuals and EC dysfunction and promote monocyte accumulation and foam cell formation in TEBVs.

TEBVs will be prepared using ECs and SMCs derived from a total of 30 patients pre-intervention and post-intervention (15-obese high-risk, 15-obese low-risk). We will examine differences in the responsiveness of the ECs to exhibit EC dysfunction and promote foam cell formation.

Procedures: All donors will be asked to sign a consent form specific for the basic-science study and will be free to withdraw from the study at any time. The volunteer will be informed that the donation is completely voluntary, that results will be maintained in confidence, and results will be presented in such a way that the volunteer's privacy is preserved. Volunteers will be compensated $25.00 for the blood drawn and informed that the maximum amount of blood that can be donated for any reason is 500 ml within a 2-month period, and that amount no more than 5 times a year. A new consent form will be filed for each donation.

50 mL of blood will be drawn from an arm vein of each donor per visit at the Duke South Durham clinic. This is a standard method for obtaining blood and is momentarily painful. There is a small risk of fainting, of some bruising (bleeding under the skin) and a rare (1 in 1000) risk of infection. To inhibit oxidation and proteolytic damage to proteins, blood is drawn into 50 ml tubes containing 0.15 M NaCl containing 1 mM Na2EDTA. Blood is centrifuged at 2000 rpm for 10 min at 5ºC, after which the supernatant is removed, placed into an autoclave, sterilized, and removed by the Safety Office. The red cells will be resuspended in phosphate buffered saline and used in the flow chamber within two hours of isolation.

High proliferative potential endothelial colony forming cells (ECFCs) will be isolated and grown according to protocols adapted from Ingram and Yoder (Blood, 2004. 104: p. 2752-2760; Blood, 2007. 109: p. 1801-1809). Blood will be diluted 1:1 with Hanks balanced salt solution (HBSS; Invitrogen, Grand Island, NY) and overlaid onto an equivalent volume of Histopaque-1077 (Sigma). The mixture will be centrifuged for 35 min at room temperature at 740 g. Buffy coat mononuclear cells will be isolated and washed 3 times in Endothelial Cell Growth Medium (EGM; Cell Applications Inc, San Diego, CA). Mononuclear cells will be resuspended in 12 mL EGM. Cells will be seeded onto 3 separate wells of a 6-well tissue culture plate coated with type 1 rat tail collagen (Corning Life Sciences, Oneonata, NY) at 37°C, 5% CO_2, in a humidified incubator. After 24 hours of culture, nonadherent cells will be removed. The media will be removed and adherent cells will be washed once with EGM medium, and EGM medium will be added to each well. Medium will be changed daily for 7 days and then every other day until cells are confluent and passaged. Individuals handling blood products and their derivatives will receive training from the Safety Office. Flow chambers and perfusion chambers used in these studies will be sterilized prior to and following procedures.

5. Data Collection

The data collected from the RESILIENCE Study may be available for use in combination with other data sources through an integrated study database. These data may include but are not limited to clinical, molecular, imaging, sensor, behavioral, and health record data. Additional datasets, including third-party data, may be included in the integrated study database. Data that are stored in the integrated study database will use a unique participant identification number. The integrated database will be maintained by Duke researchers. It is envisioned that the RESILIENCE Study data will be available to qualified researchers for exploratory analysis in the future. Qualified researchers may apply to use the data and samples from the RESILIENCE Study through applications reviewed by the Proposal Review and Publications Committee and Steering Committee. Biorepository sub-study will be submitted to the IRB as a separate new study application (Pro00106060) to be referred to for these applications.

5.1 Clinical Data

Clinical data will be collected about each participant from the Duke EHR. This will include information about the participant’s demographics, clinical history and comorbidities, medications, laboratory values

and procedures, and encounter types and frequency. In addition, height and weight information will be collected at each in-person visit.

5.2 Consent/Engagement Data

Information about the recruitment and consent process will be collected, including metrics on those who chose to progress to the consent process, degrees of completion of consent process, and other similar metrics. These data will be collected through the Mytonomy platform.

5.3 Behavioral Data

Participants will complete a battery of behavioral surveys designed to assess lifestyle habits and beliefs, as well as other parameters relevant to both weight and heart disease. The following list may be modified, but currently includes: Family Life, Activity, Sun, Health, and Eating (FLASHE)- DIET, FLASHE-Physical Activity, EARLY Self-weighing Questionnaire, Perceived Stress Scale-4 (PSS-4), Weight Self-Stigma Questionnaire (WSSQ), Reward-based Eating Drive: 5-item questionnaire (RED-X5). These surveys will be administered over the Mytonomy platform.

5.4 Biospecimen Data

Participants will give the following specimens: venous blood, saliva, and stool. Genetic analyses will be performed using the saliva samples. Metabolomic analyses will be performed using the venous blood samples. Microbiome analyses will be performed using the stool samples. For all participants, 35mL venous blood will be collected at the baseline clinic visit and for obese participants completing intervention; an additional 35mL will be collected at 6-months/end of study visit. For the basic-science sub-study who consent separately in-person, we will collect total 50 mL blood per visit (50mL at baseline and 50mL at 6-months/end of study visit).

5.5 Clinical assessments Data

5.5.1 CLIA Lab Data: The CLIA labs that will be collected are outlined in the Schedule of Assessments (Section 4.6).

5.5.2 CAC Testing Data: In half the cohort, a coronary artery calcium CT will be performed to assess the degree of coronary artery calcification (CAC score).

5.6 TRACK Intervention Data:

Data collected through the TRACK intervention will include information about participant engagement with the platform and with health coaches, and repeated weight measures using the eScale.

5.7 Fitbit Data

Data collected through Fitbits will include physical activity and sleep data.

6. Specimen Procedures/evaluations

6.1 Specimen preparation, handling, and storage

Venous blood for research purposes (not for standard clinical lab testing) will be collected by the Duke Clinical Laboratory in research tubes. All samples will be labeled with a unique RESILIENCE Study ID and barcoded. No personal health information will be used to label, track, or identify specimens. These tubes will be hand-collected by the CRC after the blood draw, and will be centrifuged, aliquoted, labelled, and stored in a -20°C freezer at the Duke South Durham Clinic. At regular intervals, these aliquots will be

transferred to the Duke Molecular Physiology Institute (DMPI). The saliva and stool specimen samples will be mailed back by individual patients directly to the DMPI.

Once received, the venous blood, saliva and stool specimens collected under this protocol will be stored in a dedicated portion of the Duke Molecular Physiology Institute (DMPI), in locked -80°C and liquid nitrogen (LN2) freezers. Newly collected blood and stool specimens will be processed, analyzed and stored in Dr. Svati Shah's (DMPI) laboratory at the Carmichael Building, 300 North Duke Street, Durham NC 27701. All samples will be labeled with thermo-safe uniquely barcoded Acquisition numbers, will be scanned into a spreadsheet freezer location template upon arrival to Dr. Shah's laboratory, and will be tracked throughout the life of the sample. Specimens collected are logged in a secure, encoded database.

6.2 Biobanking of samples

All samples will be banked for future research use. We will offer all participants the opportunity to participate in a biorepository for blood and stool storage. This biorepository sub-study aims to store subjects' plasma, DNA, and fecal samples long-term for future research. The two studies are identical in terms of sample collection. Subjects may consent for either study, both studies, or neither study and can choose to opt in or out for participation in the bio-repository. If subjects consent for both studies, an aliquot of the biorepository samples will be used for the observational trial; therefore the volume of blood and stool collected is the same if a subject consents for one study or both studies. Maximum blood drawn per participant will not exceed 85mL over any eight-week period.

DMPI will be used for long-term sample storage and sample routing. Biospecimens sent to the central biospecimen storage facility for banking will remain there until withdrawn for protocol-specified testing, unspecified future research, or until exhausted. Samples for storage for future use will be logged into the biobanking storage system using a unique identifier so their location may be tracked within the storage facility.

Please see full biorepository protocol for details which will be submitted to the IRB as a separate new study application (Pro00106060).

7. Participant Retention and Withdrawal

7.1 Strategies for Retention

Over the course of the study, a diverse set of approaches for participant engagement and retention may be employed, including but not limited to:
- Follow-up calls from a member of the study team
- Video, live-stream, or in-person engagement events
- Newsletters with opportunities to be featured or contribute content
- Feedback surveys so participants can voice their opinions
- Personalized thank you notes
- First access to research publications and other study updates
- Social media platforms such as Twitter, Facebook, YouTube, and Instagram

7.2 Discontinuation of Participants

7.2.1 Non-Compliance

At the start of the study, if participants do not return ALL THREE of their biospecimens, they will not be able to continue in the remainder of the study. During the study, participants may miss an onsite visit, not respond to a survey, not respond to the TRACK intervention or health coaches, or stop using the devices.

All efforts to regain contact will be documented and retained with the participant records. A documented effort may include multiple forms of communication. This may include, but is not limited to, the Investigator, his/her delegate, or other study personnel attempting to regain contact via the Mytonomy platform, email, mail, phone, or text.

7.2.2 Stopping study procedures or withdrawal

Participation in this study is voluntary and participants may withdraw at any time. In the event the participant chooses to withdraw, he/she will be instructed to contact the study team immediately. If a participant decides not to participate, or if they choose to drop out after the study has started, he/she may withdraw at any time without jeopardy or penalty.

During the study, participants may decide that they would like to stop some or all of the study visits or follow-up. In the event a participant requests to stop his/her involvement in the study, the participant may immediately stop all activities but will also be given several other options. These may include but are not limited to completing all or some of the in-person visits and/or active follow-up (e.g., telephone calls, email contact only) and/or passive follow-up (e.g., contact with care providers, review of health records).

If a participant expresses interest in withdrawing from the study, the study team will contact the participant and give him/her options to reduce their level of participation. This includes, but is not limited to, opting out of certain study activities. After discussions with the study team, including the Investigator or his/her delegate, if the participant decides to stop all participation in the RESILIENCE Study and withdraw entirely, the study team will inform the participant of the withdrawal process. This includes requesting information from the participant such as feedback about participation or withdrawal from the study. Participants may be asked to provide written confirmation of withdrawal. Once this information is received, further attempts to contact the participant or collect additional information will not be undertaken unless it is necessary for device retrieval. Information that has already been gathered may still be used. Participants may withdraw from the study at any time without prejudice to their care. This will not preclude the participant from re-establishing contact with the study in the future.

In the event of a participant withdrawing, the RESILIENCE Team will make every effort to help the participant return all investigational devices in their possession.

7.2.3 Replacement

Participants who do not return their initial biospecimens will not continue in the study, and they will be replaced. Participants who withdraw from the study once enrolled (and after returning the three biospecimens) will not be replaced.

7.2.4 Exiting Participants

Participants will be exited from the study for the following reasons:

- Participant becomes pregnant
- Withdrawal of consent to participate in all visits and all forms of follow-up
- Death

Participants may be exited from the study for the following reasons:

- If a participant is uncooperative or noncompliant with respect to the provisions of the protocol
- To protect the participant for safety or for administrative reasons
- Investigator determines it is in the best interest of the participant
- If a participant does not consent to continue participating in the study after being told of changes in the research that may affect him/her
- If a participant engages in misconduct, or disassembles any device used in this study

8. Analytic Considerations

8.1 Recruitment Sub-study

We will compare recruitment strategies – email vs. my chart message and ego-centric vs. altruistic messaging – and randomize in a 2x2 fashion. We will make quarterly assessments to compare the four modalities. We will compare response rates between modalities using a binomial test. If it is determined that one modality is better than another, we will drop the weaker modality. The responses will further be analyzed within defined strata. We will use an alpha spending strategy $p < 0.01$ allowing for a total of 5 comparisons for a total family wise error rate of 0.05.

8.2 Consent Sub-study

We will compare consent strategy. Again, we will make quarterly assessments. Should one strategy appear weaker than the others, we will discontinue the less effective strategy. We will use the same analytic strategy as above.

8.3 BCAA Analysis

8.3.1 Sample Size Calculation:

Based on effect sizes from our previous studies of the BCAA signature of a difference in mean (SD) levels between the two groups of 0.32 (0.95)[13], a sample size of 109 in each group would provide 80% power, indicating that we should have enough power to perform this primary analysis.

9. Assessment of Safety

9.1 Potential Risks and Benefits

For participants without obesity, there is no direct benefit for participation in this study, but the information obtained will be used in scientific research and may be helpful to the participant or others in the future. For participants with obesity, individuals may lose weight, which may lower risk for other illnesses. The list that follows includes anticipated risks. There may also be other risks that are not known at this time.

9.1.1 Privacy:

All PHI will be kept securely on Duke servers behind a firewall and with many layers of protection. Additionally, some PHI and participants' responses will be kept confidential in a HIPAA-compliant Data Server hosted in Amazon Web Services by Mytonomy. Although we will make all the efforts to keep all the study information confidential, there is a risk that someone could get unauthorized access to RESILIENCE Study data. Taking part in the study requires the use of one or more of an external study websites, mobile applications, messaging, devices, and email. Because some of these systems are developed and managed externally, there is no guarantee that they are free of risk.

9.1.2 Venipuncture:

Mild and transient local pain, mild bruising, fainting or lightheadedness, and a small risk of infection are potential consequences of the routine venipuncture that will be used for blood sample collection. A small number of individuals may have a vasovagal response to blood draws.

9.1.3 Radiation Exposure:

Individuals undergoing CAC testing will be exposed to very low degree of radiation. The radiation exposure to a participant will be detailed in the consent.

9.1.4 Exercise:

Exercise may be recommended as part of the weight loss intervention. Muscle strains, sprains, tripping, and falling can result from physical activity.

9.1.5 Women with Reproductive Potential

Because being pregnant affects diet and women, women who are pregnant or planning a pregnancy in the short-term will not be allowed to participate in this study. Women of childbearing potential will have a urine pregnancy test conducted at Duke Clinic Lab at the baseline clinic visit and it must be negative in order to participate in the study. Women who meet at least one of the following criteria will be excluded from the pregnancy screen:

- Age > 55 years old
- 12+ months since last menstrual period
- 6+ months since last menstrual period + FSH > 40 IU
- Hysterectomy
- Bilateral salpingectomy
- Bilateral oophorectomy
- Female partners
- Male partner incapable of fathering children (vasectomy NOT included)

To stay in the study, women will need to use effective methods of birth control, as outlined in the consent form. Women who become pregnant during the study will need to notify the study team immediately and be withdrawn from the study.

9.2 Monitoring

The progress of the study will be monitored to ensure that it is conducted in accordance with the protocol and applicable study and regulatory requirements. Study monitoring by the study PI will include reviewing the integrity of the clinical study data and visiting the study site periodically to observe visits and compliance with study protocol.

9.3 Event Definitions and Reporting

This is a minimal risk intervention and it is highly unlikely that we will encounter any adverse events (AE) or serious adverse events (SAE). However, our study team will review, collate, and evaluate adverse events in real-time. The PI will evaluate all adverse events within 72 hours; serious adverse events will be evaluated within 24 hours. Any study-related serious adverse event will be reported the Duke IRB within 2 weeks; all others will be included in the annual report. We will compile an aggregate AE report annually. Given the nature of the intervention, we would expect the rate of AEs to be low. In the present study, we expect that most, if any, reported injuries would result from activities unrelated to the intervention.

10. Data Management

10.1 Data Sources

All of the below data sources will be linked to our overall study data repository, which will be housed within Pedigene**.** PEDIGENE® is a flexible, integrated system for managing the research informatics needs for hundreds of studies and includes large databases containing clinical, pedigree, molecular, and sample data. It is an internationally acclaimed computer data management system designed and licensed by Duke, utilizing a multi-layered security system to ensure participant confidentiality through all phases of a family and population study. The Oracle database server is housed in a FISMA compliant data center and study data are accessible only by study personnel at Duke as well as authorized collaborators outside of Duke. Metabolomics, genetics, and microbiome analyses will be conducted per standards established for these kits.

10.1.1 EHR Data

All EHR-obtained data throughout the study duration will be linked to the main study data repository in Pedigene.

10.1.2 Consent/Engagement Data

As a part of the study, participants' personal information (such as name, email address, mobile phone number, and FHIR ID which is a unique identifying number that links participants' earlier mentioned information to Mytonomy) will be shared with the Mytonomy, Inc., who is providing services for study participants to be able to view online videos, consent and surveys. All of their responses and profile information will be kept confidential in a HIPAA-compliant Data Server hosted in Amazon Web Services by Mytonomy. This protected health information is being stored so that participants can be contacted about the study and can me assisted if they contact Mytonomy for technical help. During the study period, the website registration system will also send them automatic reminder emails and text messages to complete the various steps of the study. If it appears that participants have not completed the consent, Mytonomy may contact them in order to determine if they are having technical difficulties with the system. Participant workflow through Mytonomy's online platform and data sharing plan with Mytonomy at pre-consent and post-consent time points is outlined as demonstrated in Mytonomy workflow document. These data will then flow into the overall study data repository in Pedigene.

10.1.3 Behavioral (Self-reported) Data

Behavioral survey data will be collected and stored on the Mytonomy, Inc. platform, as above.

10.1.4 In-person Visit, Laboratory, and Imaging Data

In-person visit, laboratory, and imaging data will be collected by the CRC and entered into a study-specific RedCap database. These data will then flow into the overall study data repository in Pedigene.

10.1.5 Biospecimen Data

Data from the biospecimen analyses will be entered directly into Pedigene.

10.1.6 Device Data

Data on devices provided and returned will be collected in REDCap and TRACK platform, and will then be linked to Pedigene.

10.1.7 TRACK Intervention Data

The intervention environment (Track) is processed and stored on Heroku, an application server platform. This platform will support the following Track services: the Track database, SMS and IVR communications via Twilio, software logging, and file serving. The protections now in place are:

- Strong passwords and multi-factor authentication protect Web, shell, and console access to the Heroku dashboard.
- Application servers are inaccessible. These resources are virtualized and cannot be accessed via SSH or SFTP. The application itself is accessible only via SSL.
- No database configuration parameters (including host address, database name, password, and login) are stored in the application code or in the code repository.
- All Amazon services are protected. No identifiable information is stored on Amazon.
- Log files are available only through the Heroku dashboard. Log files do not reveal passwords, IP addresses, or PII. Identifying information is limited to the participant's masking study ID.
- SMS and IVR activity are protected by strong passwords.
- Further, logins to Heroku are severely restricted. Research staff does not have access to any computing resources.

Security-based software updated will be performed as needed, and logs will be monitored daily when the study launches. Only research staff who have received CITI Certification and IRB approval will have

access to the study database and information in the database will not be shared with anyone outside the research team.

10.2 Data Aggregation

Data collected from the RESILIENCE Study will be available for analysis alone or in combination with other data sources for broad analysis of diverse endpoints. Data may include but is not limited to site-reported data, participant-reported data, electronic health record data, insurance claims data, laboratory data, imaging data, sensor data, data from wearable and mobile devices, environmental and other publicly available data, and third-party data. Notwithstanding the above, use of data collected from the RESILIENCE Study in combination with other sources of data relating specifically to an individual, in which the combination is intended to directly identify a participant, will require review and approval by the SC and the IRB.

10.3 Data Access

It is envisioned that the RESILIENCE Study data will be available to qualified researchers for exploratory analysis in the future. Qualified external researchers may apply to use the data and samples from the RESILIENCE Study through applications reviewed by the Proposal Review and Publications Committee (Section 12.4). Biorepository sub-study will be submitted to the IRB as a separate new study application to be referred to for these applications. There will be appropriate safeguards to protect individual participant privacy.

10.4 Protocol Deviation Handling

A protocol deviation is defined as an event where the Investigator or his/her delegate did not follow, intentionally or unintentionally, the specified requirements of the study protocol. There are two types of protocol deviation categories in this study: major deviations and minor deviations.
A major deviation is defined as an occurrence that resulted in an increased risk to a participant or others that affected the rights, safety, or welfare of the participant, or the integrity of the clinical study. Examples of major deviations include, but are not limited to: failure to obtain consent prior to enrollment, or enrollment of a participant who failed to meet inclusion criteria or met exclusion criteria.
A minor protocol deviation is defined as deviating from the protocol and includes, but is not limited to: missing protocol-required tests or examinations, missed study visits, or a study visit occurring outside of the protocol-required window (early or late visit).

10.5 Record Retention

Study records that identify the subject will be kept confidential as required by law. All study data will be stored and maintained in a secure manner with physical and electronic access restrictions. Access to the above mentioned files will be limited to study-key personnel who have a need to access such information. Study data generated on Mytonomy's online platform will also be stored on it's HIPAA-compliant data server for at least six years after the study is finished. Per current Duke IRB guidelines, all the study records will be stored securely for six-years following completion of study. Please refer to the e-GRC section of the iRIS submission form for research data storage plan (RDSP).

11. Ethical Considerations

11.1 Consent

Study Informed Consent: Patients will go through the informed consent process remotely; the manner in which the informed consent information is delivered will depend on what arm they get randomized to (see Section 4.2). As part of the process, individuals will be asked questions to test their understanding of the consent material. If they answer any of these questions incorrectly, they will be prompted with the correct answer and a brief explanation. They will also have the opportunity to contact study personnel at any

point to ask questions. Once the form has been signed electronically, they will receive an electronic copy of the form, either through download/print or through email (depending on their preference).
CAC Informed Consent: Patients will go through the informed consent process for CAC testing while at their initial study visit at the South Durham clinic. This consent will be in paper form, and participants will have as much time as they need to review the form and ask any questions they have of the study coordinator. Once they have signed the form, they will receive a paper copy of the document.

11.2 Institutional Review Board

The RESILIENCE study will not begin without documented approval by Duke IRB. Over the course of the study, all amendments to the protocol and ICFs will be reviewed and approved by the IRB prior to implementation, except where necessary to eliminate apparent immediate hazards to participants

11.3 Sharing of Data

Participants' privacy will be protected by controlling access to sources of information that might potentially be used to identify the individual participants. Although qualified researchers may have access to data and samples from the RESILIENCE Study, proposals to use data and/or samples will require review and approval by the Proposal Review and Publications Committee. If approved, only the information necessary to perform the analyses specified in the use proposal will be released.
Biorepository sub-study will be submitted to the IRB as a separate new study application to be referred to for these applications. Additionally, Mytonomy may use the de-identified study records to analyze viewership of the videos and to provide information to third parties, such as other hospitals or healthcare entities, for the purpose of promoting or improving their services and conducting research.

11.4 Return of Results

The consent form will specify what will be done in the event that life-threatening or serious health information about individual participants becomes available.
Disclosure of clinical results will be completed as follows:

- Participants may receive results from designated routine CLIA-approved clinical testing from the Investigator or his/her delegate through MyChart, mail, phone, or other methods. In addition, the study team may inform the participant's primary healthcare provider about these findings.

For all of the above, participants will be notified that these results do not serve as a substitute for usual clinical care and should see their doctor at their discretion.
The potential for harm to participants in "-omics" research has been widely discussed, with the risks of discrimination in insurance and employment receiving particular emphasis. The Genetic Information Nondiscrimination Act of 2008 addresses some of these concerns; however, less often discussed but also important are other possible harms, such as familial discord or personal psychological problems that might result from a participant receiving information about their genetic profile. Such harms arise from the disclosure of information, and the privacy protections described above should prevent unplanned disclosure of individual information.

11.5 Commercial Potential

Information obtained in or generated from this project may be used to create products or material of value. Participants will not receive monetary rewards if this occurs. This will be explained in the informed consent form.

11.6 Compensation

Participants will be compensated for their participation in the RESILIENCE study. The details of compensation will be provided in the informed consent form. All participants will receive $50 after completing the first clinic visit and lab draw. Participants in the weight loss program will receive an additional $50 upon completion of the program and lab draw at 6 months. Participants with obesity who

receive a Fitbit device as part of the study will be able to keep the device at the end of the study. Participants in CAC sub-study will receive additional $50 and those in basic science sub-study will receive additional $25 at both baseline and 6-month follow-up as applicable.

12. Study Governance

12.1 Steering Committee

The Steering Committee (SC) will provide oversight of the overall design, conduct, and supervision of the RESILIENCE Study. It will adjudicate policy issues and review the progress of the study including scientific and operational data to guide the direction of the project. The SC will be responsible for approval of amendments to the study protocol. All other committees will report into the SC.

12.2 Cardiometabolic Knowledge Network

The CMKN consistent of members of the Duke community who work in the cardiometabolic space and who have an interest in participating in this and other projects in this field. The CMKN will be kept abreast of study updates and will be able to provide input that will ultimately be decided upon by the SC.

12.3 Patient Advisory Council

Our patient advisory council (PAC) has been intimately involved in the governance of the RESILIENCE project, providing feedback, insight and input on elements of study design and patient-facing engagement, recruitment and retention material. Members of the PAC are key members of the project, ensuring that our focus remains patient-centered. They have been trained, empowered and encouraged to guide all aspects of the project, from study design through dissemination of the results, playing the role of both advisors and co-designers when appropriate. To date, they have participated in the development of the project title and logo, recruitment messages, consent, and video scripts for the e-consent. They will continue to provide guidance and feedback on the project through to its completion.

12.4 Proposal Review and Publications Committee

The Proposal Review and Publications Committee will be the same as the SC for the duration of the study. After study completion, the SC may decide to create a separate committee to oversee this process. The Proposal Review and Publications Committee will be responsible for scientific and operational review and approval of all requests by qualified researchers for use of stored samples or data accrued by the RESILIENCE Study. Data and/or samples will not be released to a qualified researcher until this committee has approved the proposal and appropriate IRB approval has been obtained, when outside the scope of this protocol.

REFERENCES:

1. Obesity and overweight. World Health Organization, 2018. (Accessed October 14, 2018, at http://www.who.int/news-room/fact-sheets/detail/obesity-and-overweight.)
2. Flegal KM, Kruszon-Moran D, Carroll MD, Fryar CD, Ogden CL. Trends in Obesity Among Adults in the United States, 2005 to 2014. Jama 2016;315:2284-91.
3. Collaborators GBDO, Afshin A, Forouzanfar MH, et al. Health Effects of Overweight and Obesity in 195 Countries over 25 Years. N Engl J Med 2017;377:13-27.
4. Wilson PW, D'Agostino RB, Sullivan L, Parise H, Kannel WB. Overweight and obesity as determinants of cardiovascular risk: the Framingham experience. Arch Intern Med 2002;162:1867-72.
5. Khan SS, Ning H, Wilkins JT, et al. Association of Body Mass Index With Lifetime Risk of Cardiovascular Disease and Compression of Morbidity. JAMA Cardiol 2018;3:280-7.

6. Neeland IJ, Poirier P, Despres JP. Cardiovascular and Metabolic Heterogeneity of Obesity: Clinical Challenges and Implications for Management. Circulation 2018;137:1391-406.

7. Ortega FB, Lavie CJ, Blair SN. Obesity and Cardiovascular Disease. Circ Res 2016;118:1752-70.

8. Karczewski KJ, Snyder MP. Integrative omics for health and disease. Nat Rev Genet 2018;19:299-310.

9. MacLean PS, Rothman AJ, Nicastro HL, et al. The Accumulating Data to Optimally Predict Obesity Treatment (ADOPT) Core Measures Project: Rationale and Approach. Obesity (Silver Spring) 2018;26 Suppl 2:S6-S15.

10. Yanovski SZ, Yanovski JA. Toward Precision Approaches for the Prevention and Treatment of Obesity. JAMA 2018;319:223-4.

11. Shah SH, Crosslin DR, Haynes CS, et al. Branched-chain amino acid levels are associated with improvement in insulin resistance with weight loss. Diabetologia 2012;55:321-30.

12. Goff DC, Jr., Lloyd-Jones DM, Bennett G, et al. 2013 ACC/AHA guideline on the assessment of cardiovascular risk: a report of the American College of Cardiology/American Heart Association Task Force on Practice Guidelines. J Am Coll Cardiol 2014;63:2935-59.

Preliminary Statistical Report

Research for pErSonalized cardIovascuLar dIsease prEvention, treatmeNt, and CarE Recruitment Strategies

RESILIENCE – Aim 3

A randomized, 2x2 factorial design to determine optimal study recruitment methods of patients through modality (personal email or MyChart) by message content (altruistic or individualistic)

Protocol Date
01/22/2021

Sponsor
Duke University – Translating Duke Health Initiative

Protocol Principal Investigators
Neha Pagidipati, MD MPH

Table of Contents

1.	Overview	…………………	3
	1.1 Synopsis	…………………	3
	1.2 Exposure Groups	…………………	3
2.	Study Design	…………………	3
	2.1 Overview	…………………	3
	2.2 Randomization	…………………	3
	2.3 Data Sources	…………………	3
3.	Analysis Population and Missing Data	…………………	4
	3.1 Study Phenotypes	…………………	4
	3.2 ASCVD Risk Score Calculation	…………………	4
	3.3 Mid Study Review	…………………	4
	3.4 Missing Data	…………………	4
4.	General Methodology	…………………	4
5.	Sub Groups of Interest	…………………	4
6.	Descriptive Analyses of Patient Inclusion and Characteristics	…………………	5
7.	Primary Endpoint	…………………	5
8.	Secondary Endpoint	…………………	5
9.	Primary Endpoint Analyses	…………………	5
	9.1 Primary Endpoint: Descriptive Summaries	…………………	5
	9.2 Primary Endpoint: Inferential Analyses	…………………	6
	9.3 Primary Endpoint: Subgroup Analyses	…………………	6
10.	Secondary Endpoint Analyses	…………………	7
	10.1 Secondary Endpoint: Inferential Analyses	…………………	7
	10.2 Secondary Endpoint: Subgroup Analyses	…………………	7
12.	References	…………………	8
13.	Proposed Manuscript Tables and Figures	…………………	9-14

1. Overview

1.1 Synopsis

Aim 3 of the RESILIENCE study is to determine optimal methods of recruiting patients for clinical studies over a 6-month follow-up period from time of study invitation being sent to landing on the Mytonomy study website.

1.2 Exposure groups

The study will be a randomized 2x2 factorial design and participants will be assign to one of four recruitment arms. The factors co using personal email or MyChart to contact potential participants

2. Study Design

2.1 Overview

A randomized, 2x2 factorial design to determine optimal study recruitment methods of patients through modality (personal email or MyChart) and by message content (altruistic or individualistic).

Upon delivery of message through the respective modality and message content, participants will be given a link to access a study Mytonomy website. If a participant engages with the link they will land on the website. Landing on the study website is the outcome variable for all analysis which will be dichotomized as landing (yes vs. no) within a 6-month period from time of invitation.

The over-arching hypothesis is that, a message sent through MyChart will have more participants engaging in the Mytonomy website. Also, an altruistic message will lead to greater participant engagement compared to an individualistic message.

2.2 Randomization

Once participants are identified as eligible for the recruitment study the individuals will be assigned the recruitment strategies using block randomization of the main study phenotypes (See protocol section 4.1)

2.3 Data Sources

Potentially eligible patients will be identified using Duke Electronic Health Record (EHR) data, with additional inclusion and exclusion criteria (See protocol section 3.2). No additional data will be used to implement the analyses. Recruitment messages were sent to eligible patients between September 2019 and March 2022. Invitations to participate in the study will be distributed until the primary study's target sample size meets the requirements set in the protocol (See protocol section 3.1). A REDCap database will be created with the relevant recruitment information and assigned factorial arm. If a participant engages with the invitation's Mytonomy link the responses will be recorded by Mytonomy and delivered to the team in a .csv file upon completion of the study. The landing information in the Mytonomy file will be used as endpoints.

3. Analysis Population and Missing Data

3.1 Study Phenotypes

The population will be identified using Duke EHR and will consist of a subset of patients contacted to participate in the primary study. All inclusion and exclusion factors for the primary study are applicable to the current analyses (See protocol section 3.2).

The current study's recruitment criteria will consist of adult patients who receive care in the Duke Health System, and who fall into 1 of 3 risk group phenotypes:

#1: Patients with obesity (BMI ≥ 30) and high 10-year ASCVD risk (≥20%)
#2: Patients with obesity (BMI ≥ 30) and low 10-year ASCVD risk (<7.5%)
#3: Patients with normal weight (BMI 18-25) and low 10-year ASCVD risk (<7.5%)

3.2 ASCVD Risk Score Calculation

ASCVD risk over 10 years will be determined using the Pooled Cohorts Equation[1]. For blood pressure and cholesterol (Total Cholesterol & HDL) measures, we will calculate the empirical 1% and 99% quantiles. Any measures more extreme than these values will be truncated to the 1% and 99% respectively. For those missing any measurements (smoking, cholesterol, etc.), the worst and best case scenarios (based on the 1% and 99%) will be imputed. If a participant's ASCVD risk score is still low risk under the worst case scenario or high risk under the best case scenario, they will be categorized as such. Otherwise they will be excluded from randomization.

For the calculation of ASCVD risk score, we will determine if a participant had been or was currently on an anti-hypertensive medication from January 1, 2019 and onward.

3.3 Mid Study Review

For the Aim 1 study there will be an additional phenotype: patients with normal weight (BMI 18-25) and high 10-year ASCVD risk (≥20%). The normal weight and high risk cohort had a small pool of participants who met the above criteria. A scheduled mid study review indicated that personal email messages seemed to have a better response rate. After the mid study review, in order to optimize chances of recruiting participants with normal weight and high 10-year ASCVD risk for the Aim 1 study, participants will exclusively be contacted through personal email. Therefore, this phenotype will not be included in the final analyses for the Aim 3 recruitment study.

3.4 Missing Data

No imputation will be performed; missing data will be excluded from all denominators.

4. General Methodology

Medians, 25th and 75th percentiles, minimum, maximum and number of missings will be presented for continuous variables; the number and percentage of patients in each category will be presented for categorical variables as well as the number of missings. For all endpoints a p-value ≤0.01 will be considered statistically significant (See protocol section 8.1), to account for planned data reviews that occurred mid study. There will be no additional adjustments made for multiple comparisons within the final Aim 3 analyses. Analyses will be performed using validated SAS software (SAS Institute, Inc, Cary, NC) and R Studio. Appropriate statistical models will be used to examine the effect message and modality of recruitment messages on landing on the Mytonomy website for both the primary and secondary outcomes in the study. Point estimates (relative risks, hazard ratios) quantifying comparisons of endpoints between exposure groups will be reported together with 99% confidence intervals.

5. Sub Groups of Interest

All covariates were collected through Duke EHR at the time the recruitment invitation was sent.

#1: Age – Less than or greater than or equal to 60 years' old
#2: Sex – Male or Female
#3: Race – White, Black or African American or Other

#4: Study Phenotype - as defined in Section 3.1 of SAP.

6. Descriptive Analyses of Patient Inclusion and Characteristics

We will enumerate the number of patients participating in this study (including phenotype #4), the numbers randomized to each exposure group, and the numbers included in the final analysis. Participants are excluded from the final analysis for the following reasons:

- Patients with obesity and low ASCVD risk (4th phenotype)
- Patients missing any of the adjustment covariates
- Patients incorrectly consented
- Patients contacted more than once

Patient Characteristics: Year of invitation, risk group phenotypes (groups 1-3) as determined by above risk group criteria from EHR, Age, Gender, Race, BMI, Systolic Blood Pressure (mm Hg), Diastolic Blood Pressure (mm Hg), Total Cholesterol (mg/dL), HDL Cholesterol (mg/dL), Diabetic status, Smoker and Treated for hypertension.

Report:
Patient inclusion/exclusions are enumerated. Baseline demographic and ASCVD Risk characteristics will be described by exposure group. Table 1, Figure 1

7. Primary Endpoint

Landing on the Mytonomy website consenting website dichotomized as yes vs. no. within 6 months of receiving a study invitation. For endpoints where the response is dichotomous (binary), a robust log linear Poisson model will be used.

8. Secondary Endpoint

Time to landing on the Mytonomy website within 6 months from receiving study invitation. For time-to-event endpoints, a Cox proportional hazards model will be used.

9. Primary Endpoint Analyses

9.1 – Primary Endpoint: Descriptive Summaries

Endpoint Description: Landing on the Mytonomy website dichotomized as yes vs. no. within 6 months of receiving a study invitation.

Additional Stratification Factors: Risk group phenotypes (groups 1-3) as determined by above risk group criteria from EHR.

Statistical Tests:
The total number of landed participants (within 6 months) and the total number of participants who received an invitation will be reported for all risk group phenotypes and exposure groups. using a binomial distribution with a normal approximation (i.e., Wald confidence limits) the proportion and 99% CI for landing on the Mytonomy website (within 6 months) will be reported. Repeat report stratified by risk group phenotypes and report for risk group phenotype x modality x message.

Report:

Risk group and exposure groups will be enumerated and the proportion and 99% CI for landing will be reported. Tables 2, 3, 4, 5

9.2 – Primary Endpoint: Inferential Analyses

Endpoint Description: Landing on the Mytonomy website dichotomized as yes vs. no. within 6 months of receiving a study invitation.

Additional Covariates: Risk group phenotypes (groups 1-3) as determined by above risk group criteria from EHR.

Statistical Tests:
A robust log linear Poisson model will be used for modeling landing on the Mytonomy webpage (within 6 months) vs. not as a function of message, modality and the interaction between message x modality to find the relative risk/risk ratio (RR) of landing. The model will include the additional covariates listed above. The model will be fit using Generalized Estimating Equations (GEE) to provide a robust estimates of the covariance. A Score Test will be used to determine if there is a statistically significant interaction between message x modality.

Interaction found:
If an interaction is found, then the interaction term will be retained in the model. A significant interaction between message type and modality means that relative differences in landing rates between modalities differs by message type; and that relative differences in land rates between message types differs by modality. In the case of significant interaction, relative risks comparing modality will be reported for each message type, and vice versa. We will also refit the model with a 4-level exposure group, and report relative risks for each message/modality combination versus personal email + individualistic as the reference group.

No interaction found:
If no interaction is found, we will drop the interaction term from the model and report the main effects. In this case, RR will be reported for altruistic vs. individualistic message, and for personal mail vs. MyChart modality.

Report:
The associations between these exposure groups will be characterized by each groups as RR, the corresponding 99% confidence interval, and p-value. Table 7

9.3 – Primary Endpoint: Subgroup Analyses

Endpoint Description: Landing on the Mytonomy website dichotomized as yes vs. no. within 6 months of receiving a study invitation.

Additional Covariates: Risk group phenotypes (groups 1-3) as determined by above risk group criteria from EHR.

Sub-groups: Age at randomization, sex, race and risk group phenotypes.

Statistical Tests:
A robust log linear Poisson regression model for the landing on the Mytonomy webpage vs not landing (within 6 months) will be modeled as a function of the exposure groups (main effects), subgroup and the interaction between exposure groups x subgroup.

Note: Risk group phenotype will be included as an adjustment covariate in models for subgroup analyses, except when risk group phenotype is the subgroup factor of interest.

Report:
The associations will be characterized by RR (99% CI) for each exposure group comparison, within each subgroup. The interaction p-value will be reported to assess whether the RR comparing exposure groups differs across levels of each subgroup. Table 8, Figure 2

10. Secondary Endpoint Analyses

10.1 – Secondary Endpoint: Inferential Analyses

Endpoint Description: Time to landing on the Mytonomy website within 6 months from receiving study invitation

Additional Covariates: Risk group phenotypes (groups 1-3) as determined by above risk group criteria from EHR.

Statistical Tests:
A Cox proportional hazard model will be used to examine the association between time from study invitation to landing as a function of message, modality, and the interaction between message x modality. The model will adjust for the additional covariates listed above. The proportional hazards assumption will be evaluated using Schoenfeld residuals. If the interaction between message x modality is not statistically significant, then a simplified model will be fit just including the main effects.

Report:
The impact of message and modality on landing will be characterized using the HR and corresponding 99% CI. Table 9, Figure 3

10.2 – Secondary Endpoint: Subgroup Analyses

Endpoint Description: Time to landing on the Mytonomy website within 6 months from receiving study invitation

Sub-groups: Age at randomization, sex, race and risk group phenotypes

Additional Covariates: Risk group phenotypes (groups 1-3) as determined by above risk group criteria from EHR.

Statistical Tests: Cox proportional hazard model will be used to examine the association between time to landing as a function of the exposure groups (main effects), subgroup and the interaction of exposure groups x subgroup.

Note: Risk group phenotypes will be included as an adjustment covariate in models for subgroup analyses, except when risk group phenotype is the subgroup factor of interest.

Report:
The associations will be characterized by RR (99% CI) for each exposure group comparison, within each subgroup. The interaction p-value will be reported to assess whether there are differences in response to exposure groups by subgroup levels. Table 10, Figures 4, 5, 6, 7

References:

Goff DC, Jr., Lloyd-Jones DM, Bennett G, et al. 2013 ACC/AHA guideline on the assessment of cardiovascular risk: a report of the American College of Cardiology/American Heart Association Task Force on Practice Guidelines. J Am Coll Cardiol 2014;63:2935-59.

Appendix I

Preliminary Tables

and

Figures

Table 1

Baseline characteristics stratified by message and modality (N=15,376)

		Modality		Message	
**Baseline Characteristics**	**All Participants (N=15,376)**	**Email (N=7,737)**	**MyChart (N=7,639)**	**Altruistic (N=7,682)**	**Individualistic (N=7,694)**
Study Phenotypes					
Obese High Risk	6,667 (43.4%)	3,420 (44.2%)	3,247 (42.5%)	3,329 (43.3%)	3,338 (43.4%)
Obese Low Risk	4,055 (26.4%)	2,008 (26.0%)	2,047 (26.8%)	2,026 (26.4%)	2,029 (26.4%)
Non-Obese Low Risk	4,654 (30.3%)	2,309 (29.8%)	2,345 (30.7%)	2,327 (30.3%)	2,327 (30.2%)
Year of Invitation					
2020	4,651 (30.2%)	2,305 (29.8%)	2,346 (30.7%)	2,325 (30.3%)	2,326 (30.2%)
2021	8,575 (55.8%)	4,260 (55.1%)	4,315 (56.5%)	4,290 (55.8%)	4,285 (55.7%)
2022	2,150 (14.0%)	1,172 (15.1%)	978 (12.8%)	1,067 (13.9%)	1,083 (14.1%)
**Demographics**					
Age (years)					
>60	8,240 (53.6%)	4,140 (53.5%)	4,100 (53.7%)	4,070 (53.0%)	4,170 (54.2%)
≤60	7,136 (46.4%)	3,597 (46.5%)	3,539 (46.3%)	3,612 (47.0%)	3,524 (45.8%)
Gender					
Female	9,309 (60.5%)	4,644 (60.0%)	4,665 (61.1%)	4,649 (60.5%)	4,660 (60.6%)
Male	6,067 (39.5%)	3,093 (40.0%)	2,974 (38.9%)	3,033 (39.5%)	3,034 (39.4%)
Race					
White	9,346 (60.8%)	4,721 (61.0%)	4,625 (60.5%)	4,667 (60.8%)	4,679 (60.8%)
Black or African American	4,452 (29.0%)	2,221 (28.7%)	2,231 (29.2%)	2,206 (28.7%)	2,246 (29.2%)
Other	1,203 (7.8%)	619 (8.0%)	584 (7.6%)	626 (8.1%)	577 (7.5%)
Not Reported/Declined	375 (2.4%)	176 (2.3%)	199 (2.6%)	183 (2.4%)	192 (2.5%)
BMI					
Median (25th, 75th)	32.2 (24.2, 36.4)	32.2 (24.3, 36.5)	32.2 (24.2, 36.4)	32.2 (24.2, 36.5)	32.2 (24.2, 36.4)
Min, Max	18.0, 5042	18.0, 96.6	18.0, 5042	18.0, 5042	18.0, 96.6
Missing	0	0	0	0	0
Systolic Blood Pressure (mm Hg)					
Median (25th, 75th)	128.0 (117.0, 140.0)	129.0 (117.0, 141.0)	128.0 (116.0, 140.0)	128.0 (117.0, 140.0)	128.0 (117.0, 141.0)
Min, Max	73.0, 250.0	73.0, 238.0	78.0, 250.0	82.0, 250.0	73.0, 238.0
Missing	84	39	45	38	46
Diastolic Blood Pressure (mm Hg)					
Median (25th, 75th)	78.0 (71.0, 84.0)	79.0 (72.0, 84.0)	78.0 (71.0, 84.0)	78.0 (71.0, 84.0)	78.0 (72.0, 84.0)
Min, Max	8.0, 150.0	8.0, 147.0	41.0, 150.0	8.0, 150.0	38.0, 147.0
Missing	84	39	45	38	46
Treated for Hypertension	7,557 (49.3%)	3,799 (49.2%)	3,758 (49.3%)	3,783 (49.4%)	3,774 (49.2%)
Total Cholesterol (mg/dL)					
Median (25th, 75th)	187.0 (159.0, 215.0)	187.0 (159.0, 215.0)	187.0 (159.0, 216.0)	187.0 (160.0, 215.0)	186.0 (159.0, 216.0)
Min, Max	54.0, 588.0	54.0, 460.0	72.0, 588.0	54.0, 442.0	54.0, 588.0
Missing	2421	1211	1210	1228	1193
HDL Cholesterol (mg/dL)					
Median (25th, 75th)	49.0 (40.0, 61.0)	49.0 (39.0, 61.0)	49.0 (40.0, 62.0)	49.0 (39.0, 61.0)	49.0 (40.0, 62.0)
Min, Max	12.0, 153.0	14.0, 145.0	12.0, 153.0	13.0, 145.0	12.0, 153.0
Missing	2426	1211	1215	1231	1195

		Modality		Message	
**Baseline Characteristics**	**All Participants (N=15,376)**	**Email (N=7,737)**	**MyChart (N=7,639)**	**Altruistic (N=7,682)**	**Individualistic (N=7,694)**
Diabetic	4,853 (31.6%)	2,447 (31.6%)	2,406 (31.5%)	2,442 (31.8%)	2,411 (31.3%)
Smoker	1,307 (8.5%)	670 (8.7%)	637 (8.3%)	621 (8.1%)	686 (8.9%)

Generated from dcri/study/tdh_resilience/tables/aim3/Base_1/baseline.rtf/messagexmodality.sas on 07AUG2023 at 11:54

Table 2.
Overall landing rates.

Cohort/Message/Modality	Not Landed	Landed	Total	Rate (99% CI)
Overall	14,156	1,220	15,376	0.079 (0.074, 0.085)
Obese High Risk	6,198	469	6,667	0.07 (0.062, 0.078)
Obese Low Risk	3,647	408	4,055	0.101 (0.088, 0.113)
Non-Obese Low Risk	4,311	343	4,654	0.074 (0.064, 0.084)
Altruistic	7,048	634	7,682	0.083 (0.074, 0.091)
Individualistic	7,108	586	7,694	0.076 (0.068, 0.084)
Email	6,969	768	7,737	0.099 (0.091, 0.108)
MyChart	7,187	452	7,639	0.059 (0.052, 0.066)

Generated from dcri/study/tdh_resilience/tables/aim3/landing/update/landing V5.SAS on 18SEP2023 at 12:00

Table 3.
Landing rates for obese high risk cohort.

Message/Modality	Not Landed	Landed	Total	Rate (99% CI)
Email	3,114	306	3,420	0.089 (0.077, 0.102)
MyChart	3,084	163	3,247	0.05 (0.04, 0.06)
Altruistic	3,085	244	3,329	0.073 (0.062, 0.085)
Individualistic	3,113	225	3,338	0.067 (0.056, 0.079)

Generated from dcri/study/tdh_resilience/tables/aim3/landing/update/landing V5.SAS on 18SEP2023 at 12:00

Table 4.
Landing rates for obese low risk cohort.

Message/Modality	Not Landed	Landed	Total	Rate (99% CI)
Email	1,741	267	2,008	0.133 (0.113, 0.152)
MyChart	1,906	141	2,047	0.069 (0.054, 0.083)
Altruistic	1,826	200	2,026	0.099 (0.082, 0.116)
Individualistic	1,821	208	2,029	0.103 (0.085, 0.12)

Generated from dcri/study/tdh_resilience/tables/aim3/landing/update/landing V5.SAS on 18SEP2023 at 12:00

Table 5.
Landing rates for non-obese low risk cohort.

Message/Modality	Not Landed	Landed	Total	Rate (99% CI)
Email	2,114	195	2,309	0.084 (0.07, 0.099)
MyChart	2,197	148	2,345	0.063 (0.05, 0.076)
Altruistic	2,137	190	2,327	0.082 (0.067, 0.096)
Individualistic	2,174	153	2,327	0.066 (0.053, 0.079)

Generated from dcri/study/tdh_resilience/tables/aim3/landing/update/landing V5.SAS on 18SEP2023 at 12:00

Table 6.
Landing rate for each combination of cohort x modality x message.

Message/Modality	Not Landed	Landed	Total	Rate (99% CI)
Obese High Risk, Email, Altruistic	1,554	164	1,718	0.095 (0.077, 0.114)
Obese High Risk, Email, Individualistic	1,560	142	1,702	0.083 (0.066, 0.101)
Obese High Risk, MyChart, Altruistic	1,531	80	1,611	0.05 (0.036, 0.064)
Obese High Risk, MyChart, Individualistic	1,553	83	1,636	0.051 (0.037, 0.065)
Obese Low Risk, Email, Altruistic	878	124	1,002	0.124 (0.097, 0.151)
Obese Low Risk, Email, Individualistic	863	143	1,006	0.142 (0.114, 0.171)
Obese Low Risk, MyChart, Altruistic	948	76	1,024	0.074 (0.053, 0.095)
Obese Low Risk, MyChart, Individualistic	958	65	1,023	0.064 (0.044, 0.083)
Non-Obese Low Risk, Email, Altruistic	1,048	107	1,155	0.093 (0.071, 0.115)
Non-Obese Low Risk, Email, Individualistic	1,066	88	1,154	0.076 (0.056, 0.096)
Non-Obese Low Risk, MyChart, Altruistic	1,089	83	1,172	0.071 (0.052, 0.09)
Non-Obese Low Risk, MyChart, Individualistic	1,108	65	1,173	0.055 (0.038, 0.073)

Generated from dcri/study/tdh_resilience/tables/aim3/landing/update/landing V5.SAS on 18SEP2023 at 12:00

Table 7.
Robust log linear Poisson regression for the association of message and modality of recruitment method of landing on the Mytonomy website adjusted by study phenotypes.

	RR (99% CI)	P-Value
**Message**		
Altruistic	1.08 (0.94, 1.25)	0.15
Individualistic	Ref	
**Modality**		
Email	1.68 (1.45, 1.95)	<0.001
MyChart	Ref	
*Interaction p-value not significant		

Generated from dcri/study/tdh_resilience/tables/aim3/primary/table 7 V4.SAS on 18SEP2023 at 11:56

Table 8.

Robust log linear Poisson regression for the association of the exposure groups, sub groups and the interaction (exposure groups x subgroups) of landing on the Mytonomy website adjusted by study phenotypes.

	Message Altruistic vs. Individualistic RR (99% CI)	Modality Email vs. MyChart RR (99% CI)
**Age (years)**		
≤60	1.21 (1, 1.48)	1.51 (1.24, 1.85)
>60	0.95 (0.77, 1.16)	1.92 (1.55, 2.38)
Interaction p-value	0.025	0.036
**Race**		
White	1.08 (0.92, 1.28)	1.56 (1.32, 1.85)
Black or African American	1.02 (0.74, 1.4)	1.97 (1.41, 2.75)
Other	1.38 (0.76, 2.51)	2.48 (1.29, 4.75)
Interaction p-value	0.498	0.064
**Gender**		
Male	1.11 (0.87, 1.43)	1.81 (1.39, 2.36)
Female	1.07 (0.9, 1.27)	1.63 (1.37, 1.94)
Interaction p-value	0.723	0.383
**Study Phenotypes**		
Obese Low Risk	0.96 (0.76, 1.23)	1.93 (1.49, 2.49)
Obese High Risk	1.09 (0.86, 1.37)	1.78 (1.4, 2.27)
Non-obese Low Risk	1.24 (0.95, 1.63)	1.34 (1.02, 1.75)
Interaction p-value	0.193	0.032

Generated from dcri/study/tdh_resilience/tables/aim3/primary/table 8 V3.SAS on 18SEP2023 at 11:56

Table 9.
Cox proportional hazard model for the time to landing by message and modality adjusted by study phenotype.

	Number Landed	RR (99% CI)	P-Value
**Message**			
Altruistic	634	1.08 (0.93, 1.26)	0.16
Individualistic	586	Ref	
**Modality**			
Email	768	1.73 (1.48, 2.01)	<0.001
MyChart	452	Ref	

*Interaction p-value not significant

Generated from dcri/study/tdh_resilience/tables/aim3/secondary/Table9.SAS on 14SEP2023 at 16:33

Supplementary figure for table 9– ZPH plot for proportional hazard violation

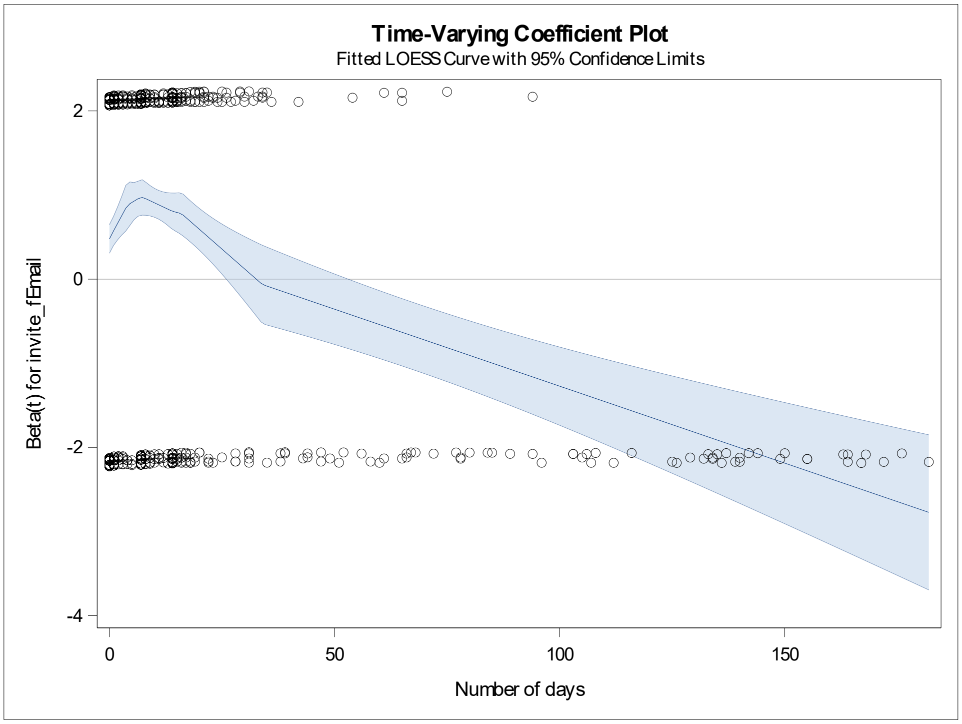

Table 10.

Cox proportional hazard model for the time to landing on Mytonomy website by exposure groups, sub groups and the interactions (exposure groups x subgroups) adjusted by study phenotype.

	Message Altruistic vs. Individualistic RR (99% CI)	Modality Email vs. MyChart RR (99% CI)
**Age (years)**		
≤60	1.23 (1, 1.51)	1.54 (1.25, 1.9)
>60	0.95 (0.77, 1.17)	1.98 (1.58, 2.47)
Interaction p-value	0.024	0.036
**Race**		
White	1.09 (0.91, 1.29)	1.6 (1.34, 1.91)
Black or African American	1.02 (0.73, 1.4)	2.02 (1.43, 2.85)
Other	1.39 (0.75, 2.56)	2.53 (1.3, 4.91)
Interaction p-value	0.507	0.092
**Gender**		
Male	1.12 (0.86, 1.45)	1.85 (1.41, 2.43)
Female	1.07 (0.9, 1.28)	1.68 (1.4, 2.02)
Interaction p-value	0.742	0.447
**Study Phenotypes**		
Obese Low Risk	0.96 (0.75, 1.24)	2.02 (1.54, 2.64)
Obese High Risk	1.09 (0.86, 1.38)	1.82 (1.42, 2.34)
Non-obese Low Risk	1.25 (0.95, 1.65)	1.35 (1.02, 1.79)
Interaction p-value	0.204	0.023

Generated from dcri/study/tdh_resilience/tables/aim3/secondary/Table10.SAS on 27SEP2023 at 16:12

Figure 1. Consort diagram of participant movement through the recruitment study.

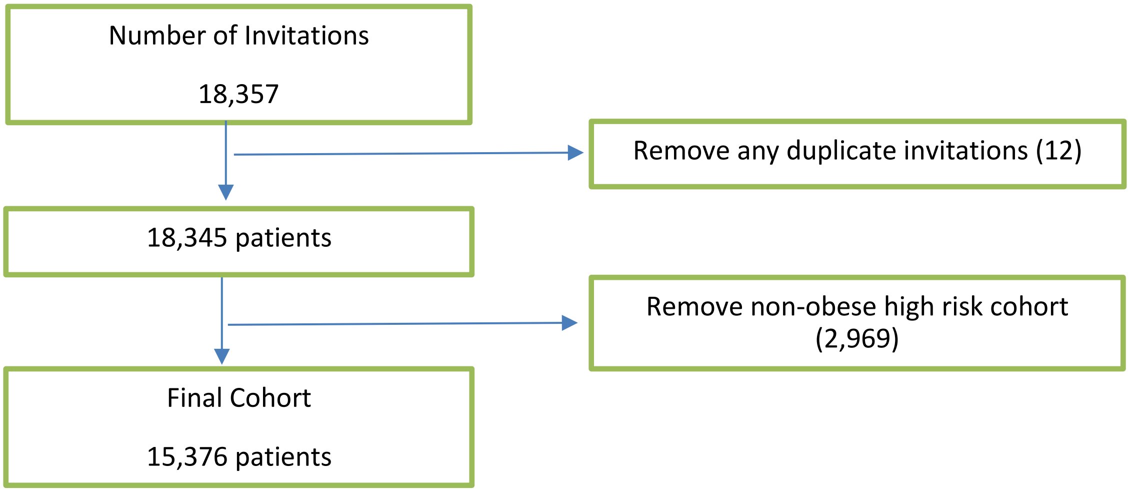

Figure 2. Forest plot of RR for landing of exposure group by subgroups.

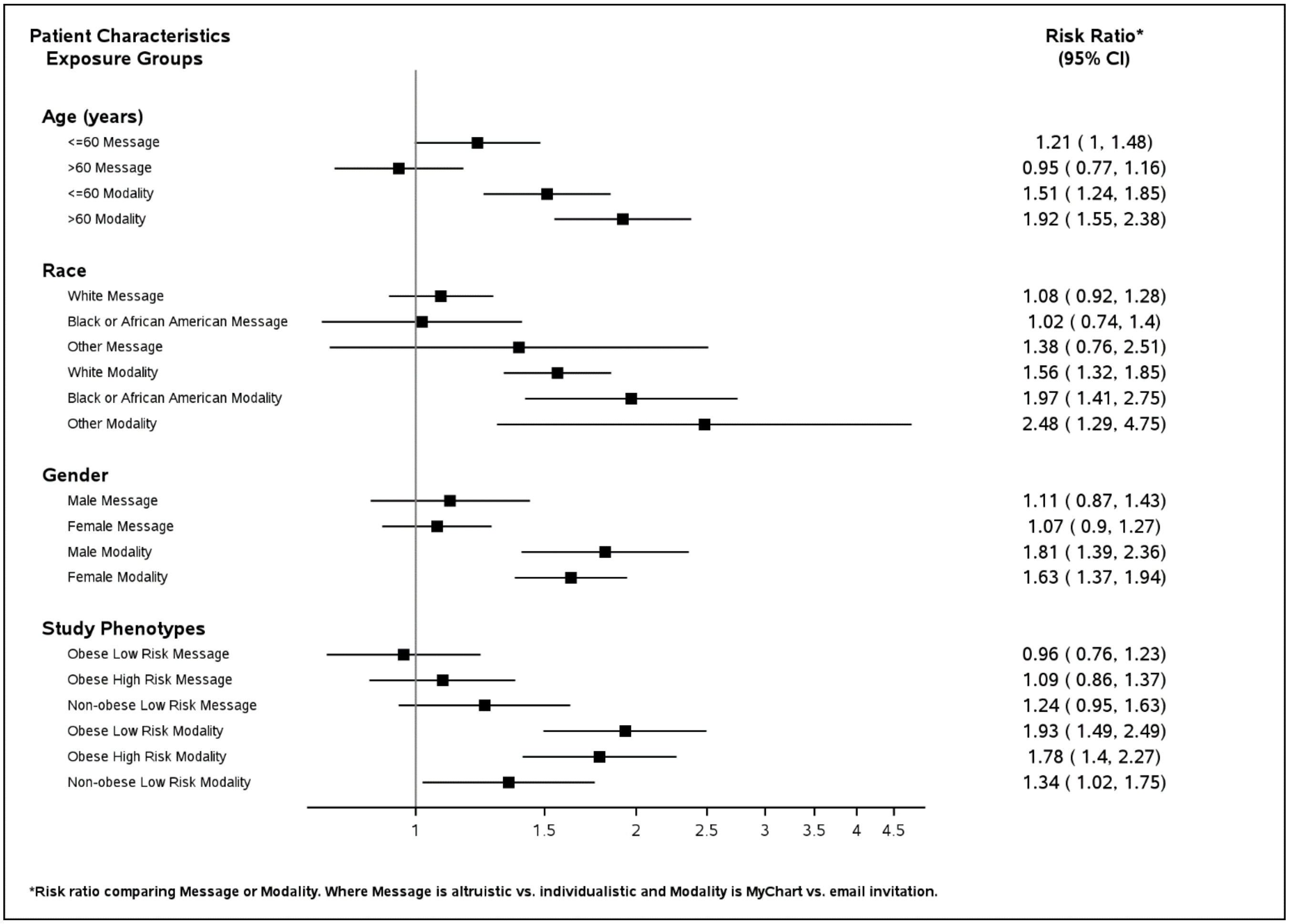

Figure 3. Cumulative Incidences for landed vs not landed of exposure group.

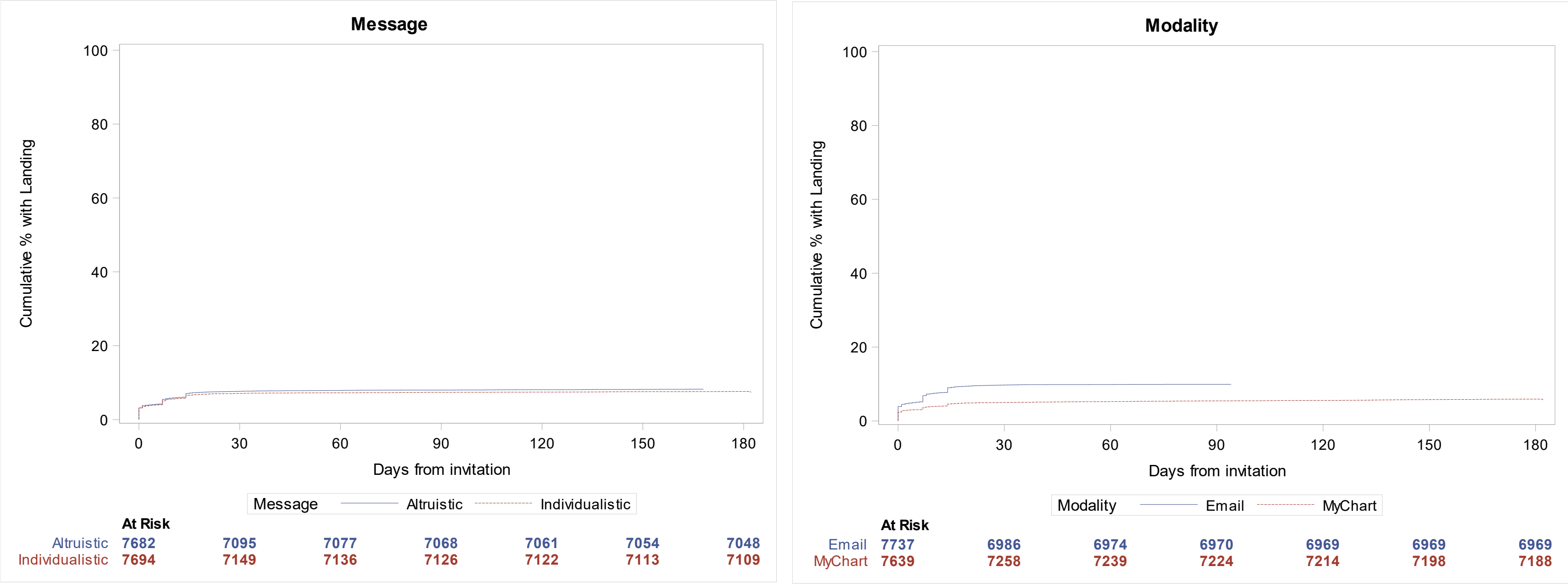

Figure 4. Cumulative Incidences for landed vs not landed of exposure group by gender.

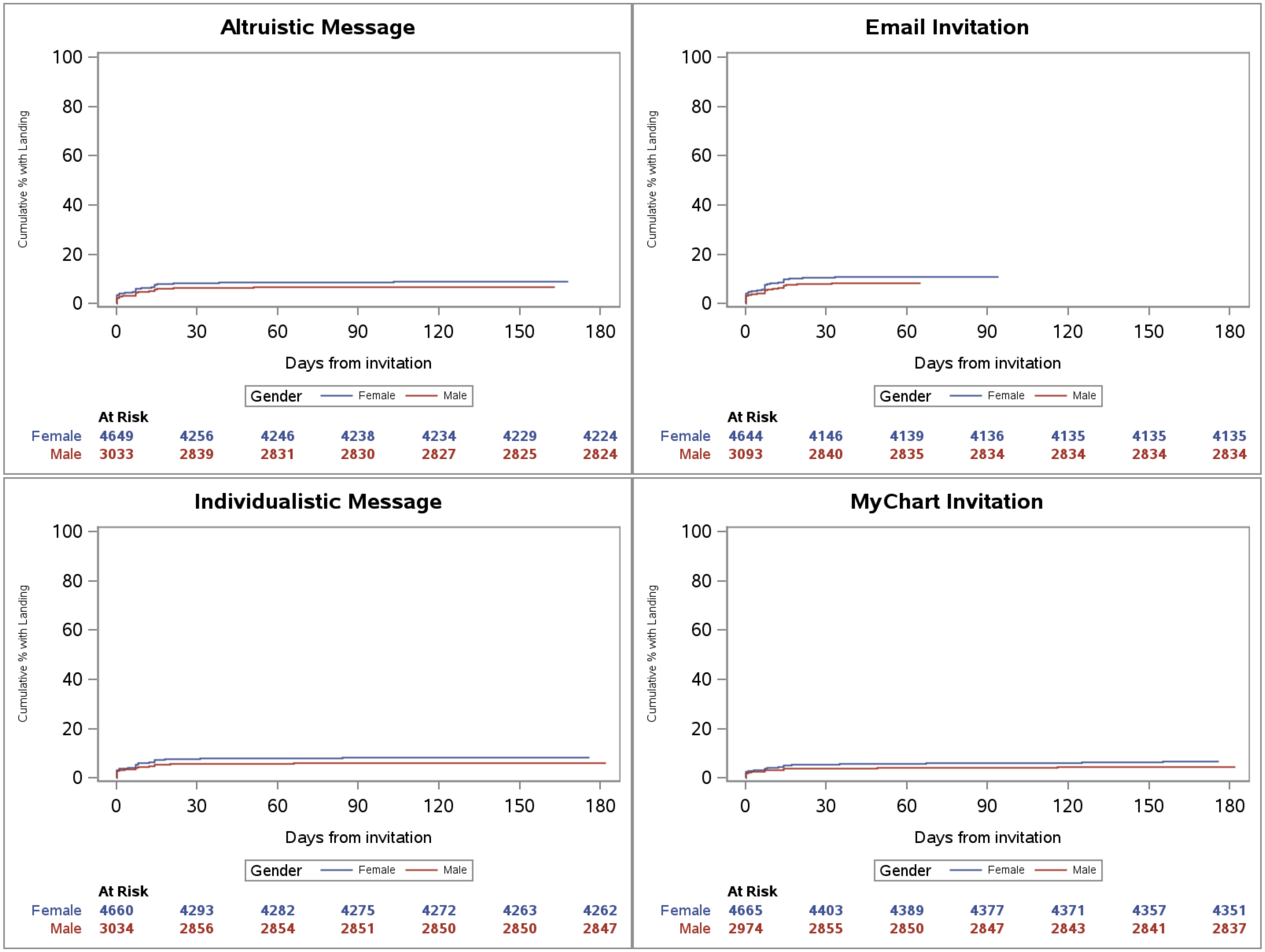

Figure 5. Cumulative Incidences for landed vs not landed of exposure group by age.

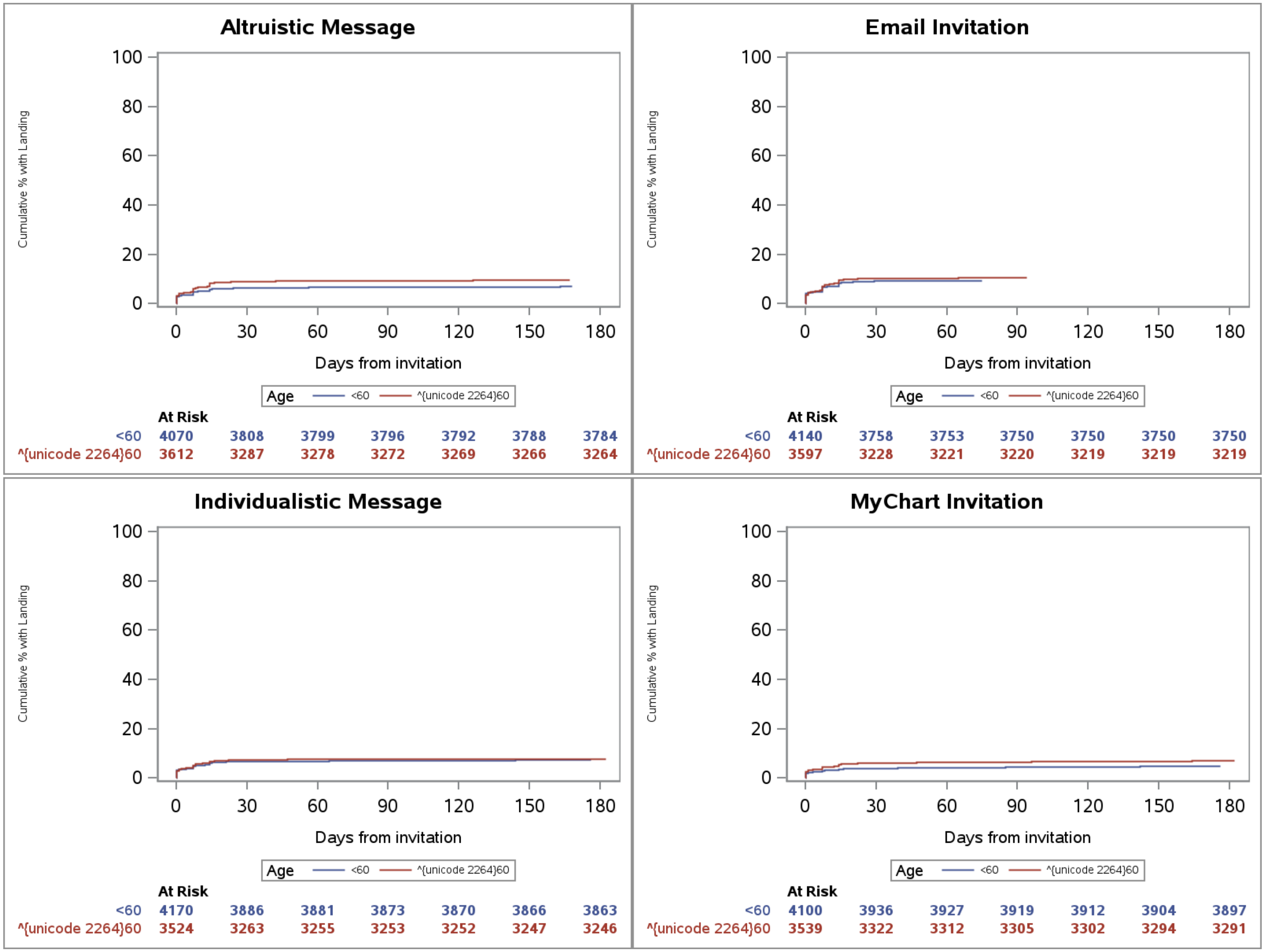

Figure 6. Cumulative Incidences for landed vs not landed of exposure group by race.

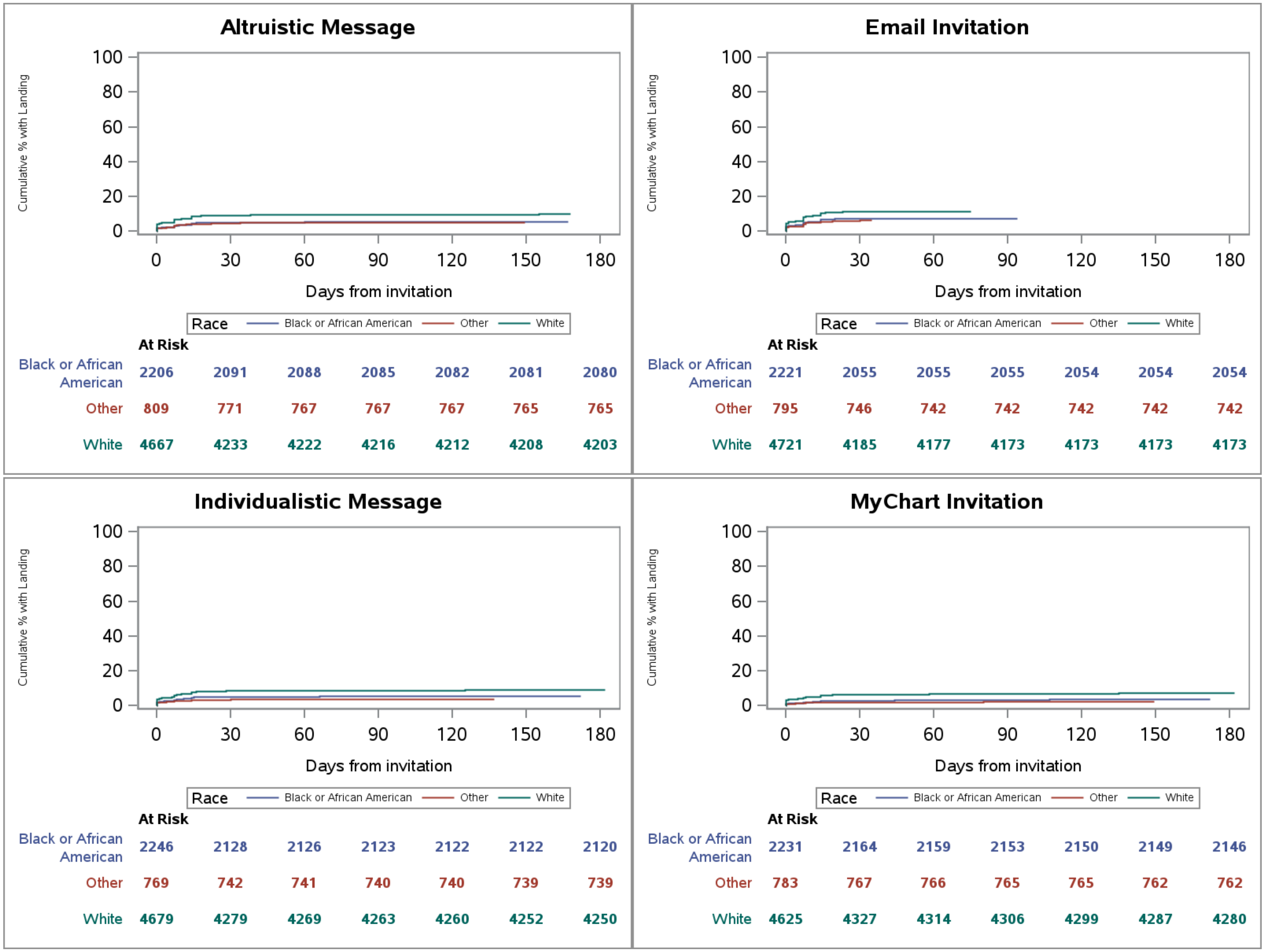

Figure 7. Cumulative Incidences for landed vs not landed of exposure group by phenotype.

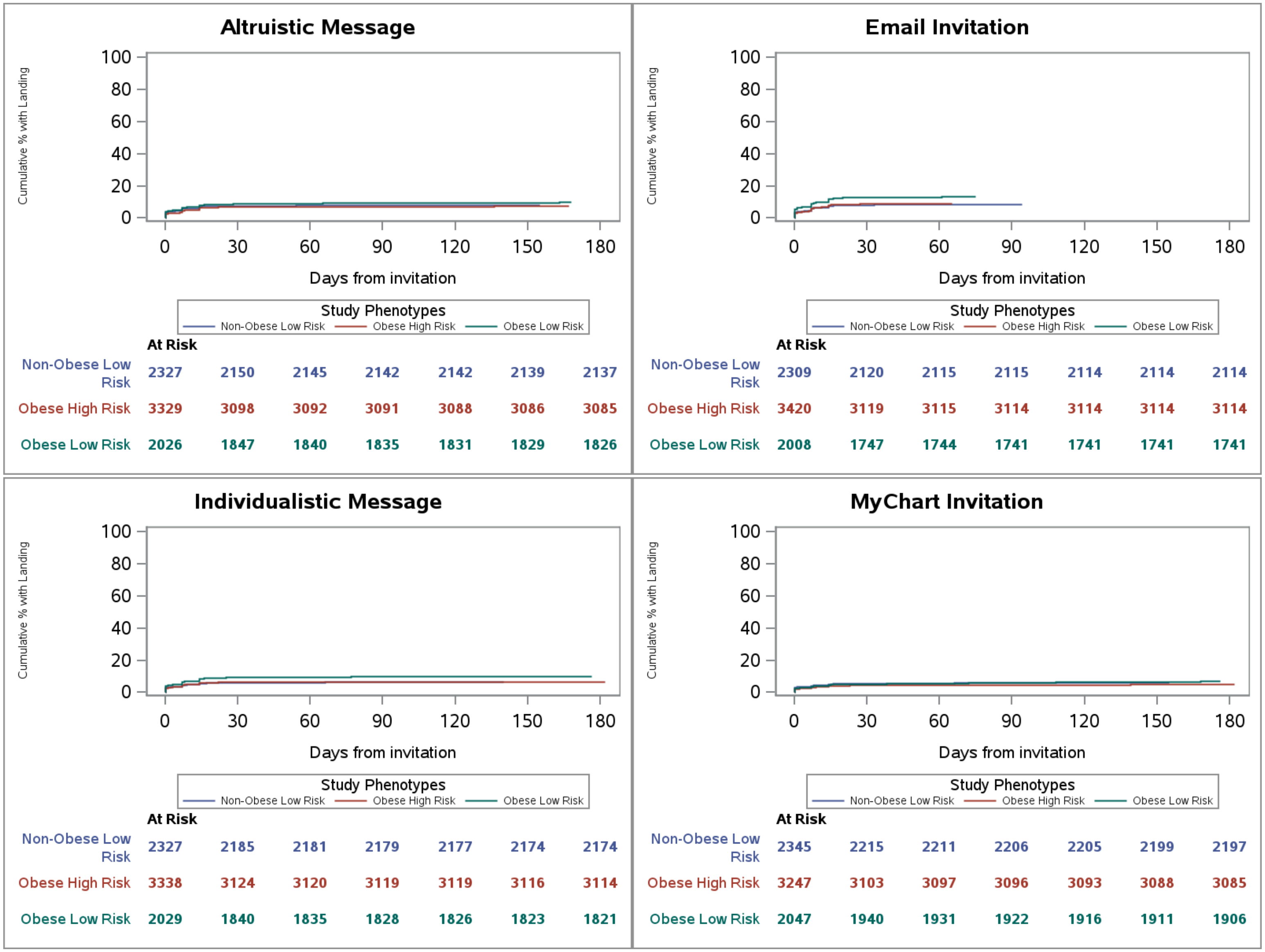

Supplementary Figures

Supplementary for figure 4. Cumulative Incidences for landed vs not landed of exposure group by gender.

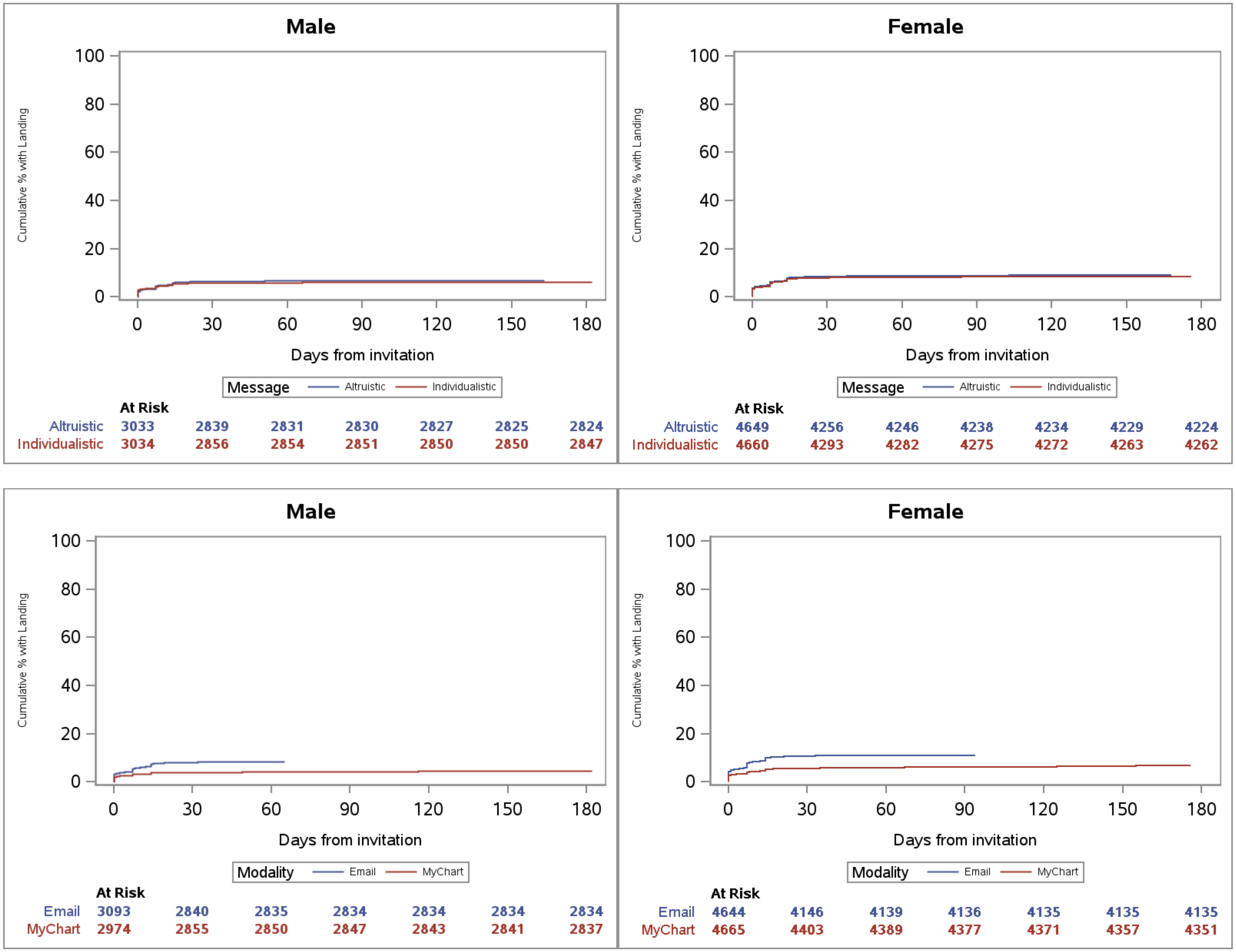

Supplementary for figure 5. Cumulative Incidences for landed vs not landed of exposure group by age.

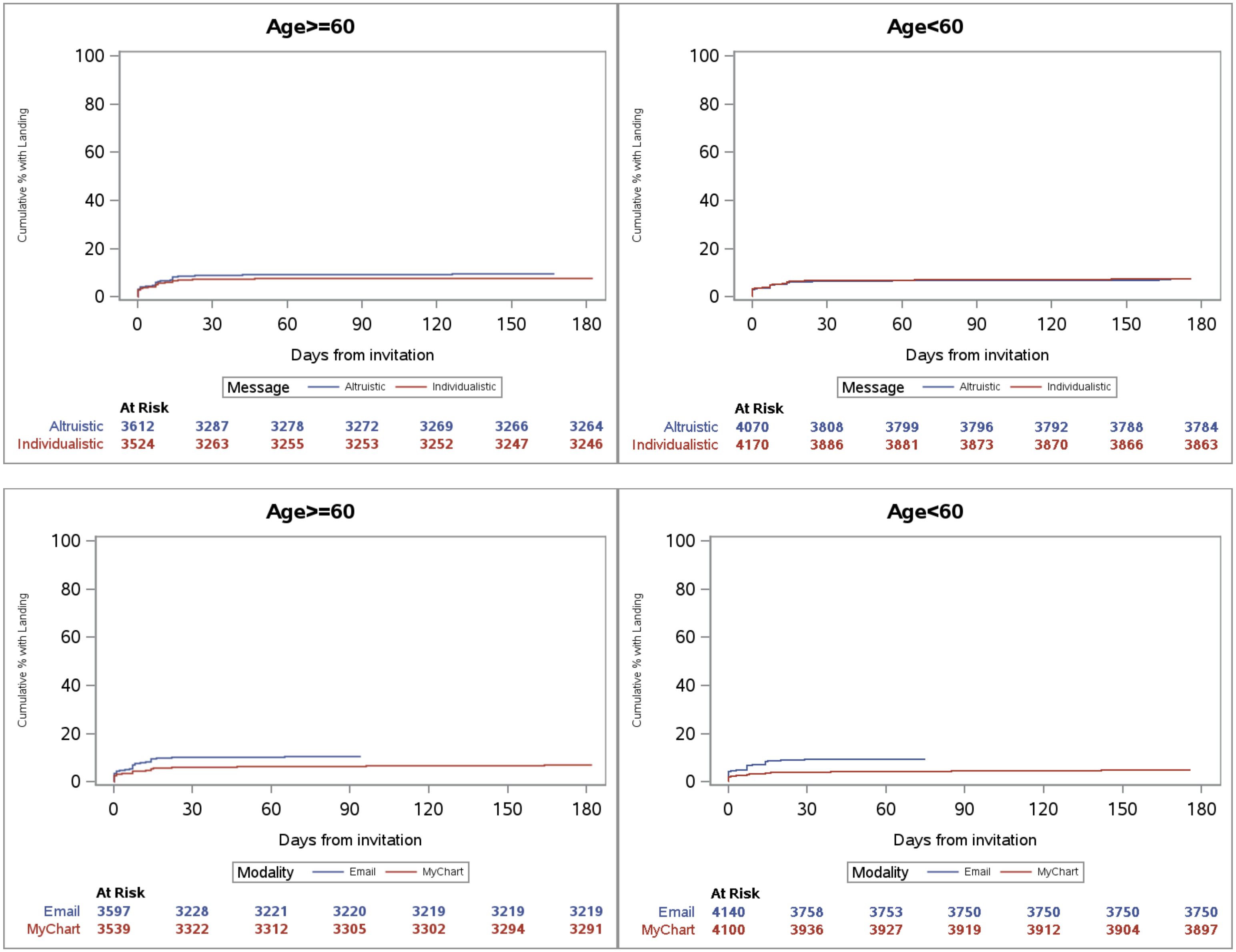

Supplementary for figure 6. Cumulative Incidences for landed vs not landed of exposure group by race.

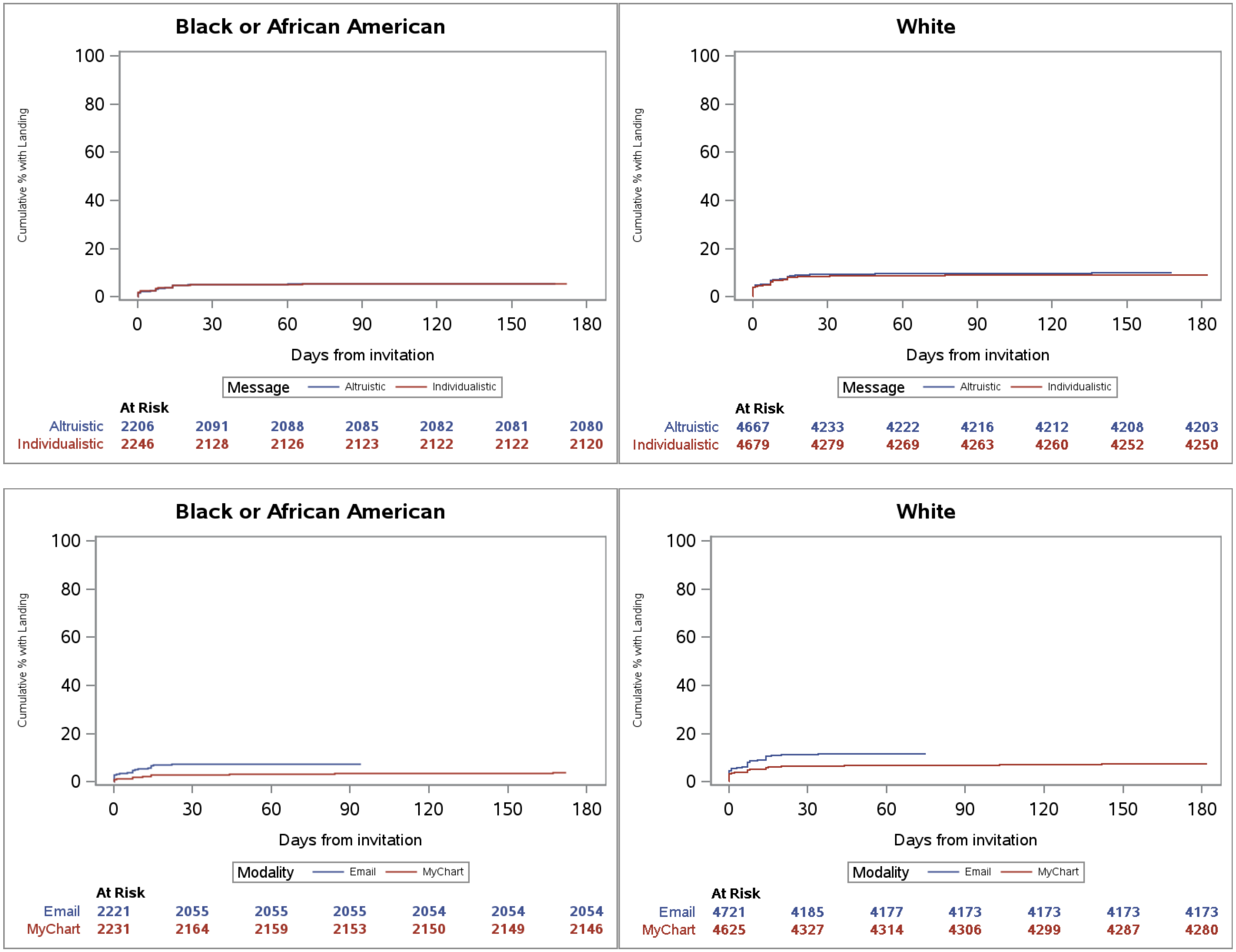

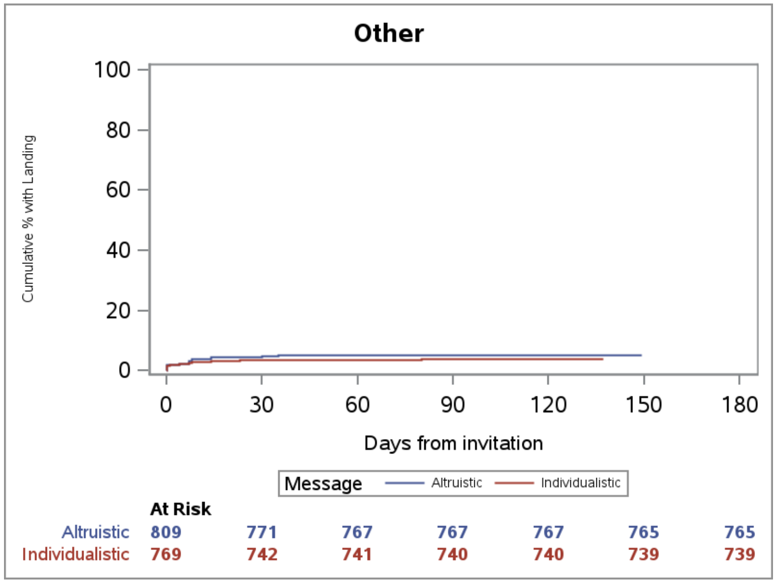
Other
Cumulative % with Landing
100
80
60
40
20
0
0
30
60
90
120
150
180
Days from invitation
Message
Altruistic
Individualistic
At Risk
Altruistic 809 771 767 767 767 765 765
Individualistic 769 742 741 740 740 739 739

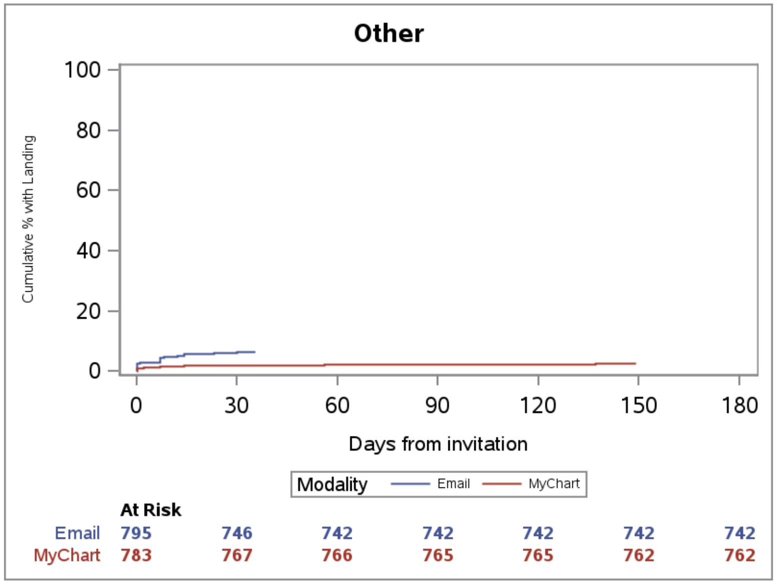
Other
Cumulative % with Landing
100
80
60
40
20
0
0
30
60
90
120
150
180
Days from invitation
Modality
Email
MyChart
At Risk
Email 795 746 742 742 742 742 742
MyChart 783 767 766 765 765 762 762

Supplementary for figure 7. Cumulative Incidences for landed vs not landed of exposure group by phenotype.

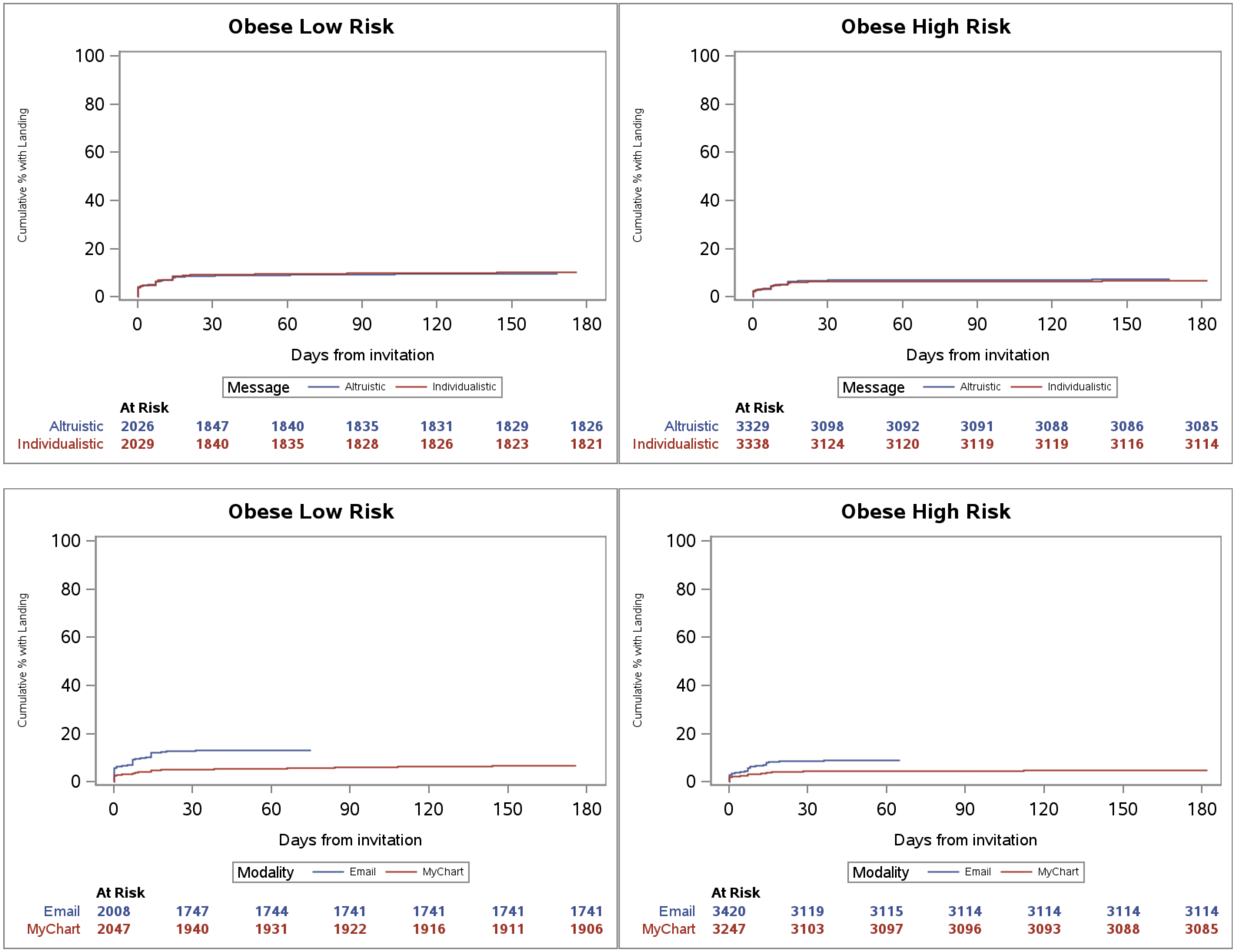

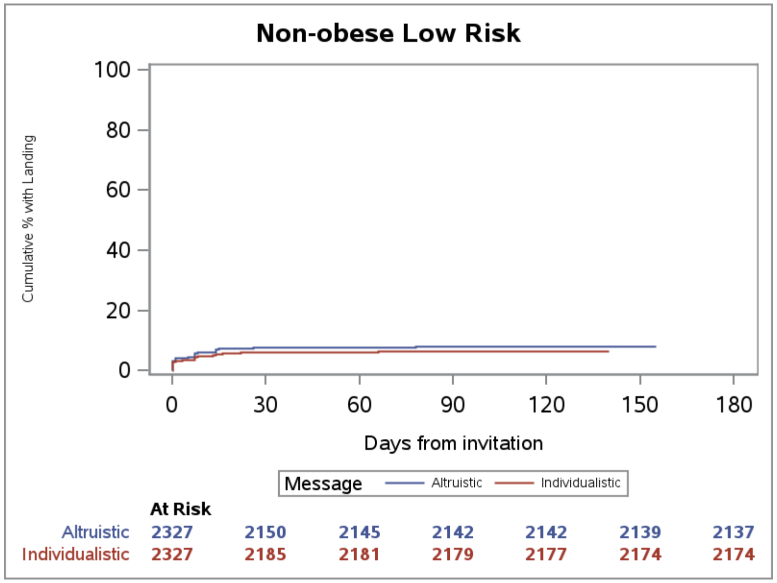
Non-obese Low Risk
Cumulative % with Landing
100
80
60
40
20
0
0 30 60 90 120 150 180
Days from invitation
Message
Altruistic
Individualistic
At Risk
Altruistic 2327 2150 2145 2142 2142 2139 2137
Individualistic 2327 2185 2181 2179 2177 2174 2174

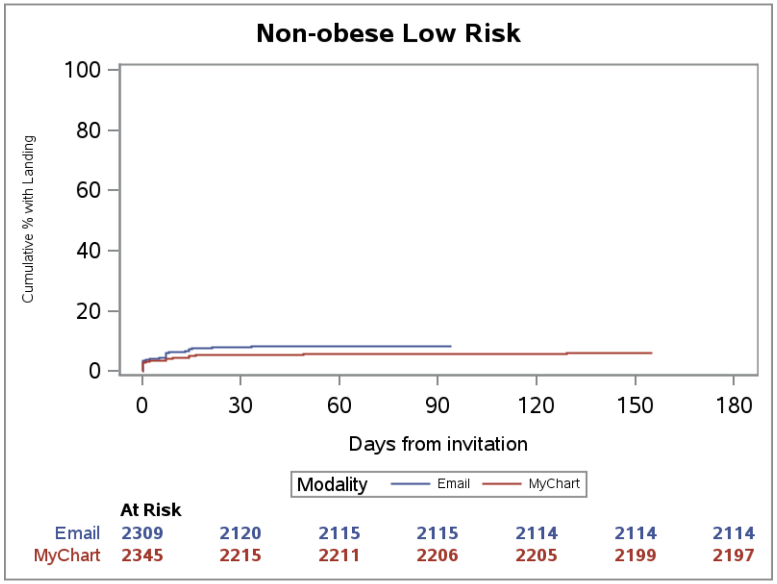
Non-obese Low Risk
Cumulative % with Landing
100
80
60
40
20
0
0 30 60 90 120 150 180
Days from invitation
Modality
Email
MyChart
At Risk
Email 2309 2120 2115 2115 2114 2114 2114
MyChart 2345 2215 2211 2206 2205 2199 2197

461

History of Changes to this Document	
**Date**	**Description of Change(s)**
8/9/2023	Preliminary report drafted
8/31/2023	Preliminary report drafted V1.1 – update tables 2-7
9/18/2023	Updates to tables and figures
9/26/2023	Updates to tables and figures
