# Supplementary material for: Messaging Modality and Content for Recruitment of Research Participants: A Randomized Clinical Trial
Source: JAMA Netw Open. 2026 May 22;9(5):e2614046. doi: 10.1001/jamanetworkopen.2026.14046 (PMC13197867; doi:10.1001/jamanetworkopen.2026.14046)
Supplement: Supplement 2. — eTable 1. Message Content eTable 2. Exploratory Subgroup Analysis of Primary Outcome of Potential Participant Engagement [file jamanetwopen-e2614046-s002.pdf]

Supplemental Online Content

Gouda P, Glover L, Kenjale A, et al. Messaging modality and content for recruitment of research participants: a randomized clinical trial. *JAMA Netw Open.* 2026;9(5):e2614046. doi:10.1001/jamanetworkopen.2026.14046

eTable 1. Message Content

eTable 2. Exploratory Subgroup Analysis of Primary Outcome of Potential Participant Engagement

This supplemental material has been provided by the authors to give readers additional information about their work.

© 2026 Gouda P et al. *JAMA Network Open*.

eTable 1. Message Content

**Eligible Participants with BMI≥30 kg/m²**		**Eligible Participants with BMI<30 kg/m²**	
Altruistic	**Individualistic**	**Altruistic**	**Individualistic/Science-centric**
Everyday millions of people in the US struggle with their weight. Some respond to weight loss treatments while others do not. Likewise, some develop heart disease while others do not. We are doing the RESILIENCE study to understand more about these differences so that we can develop better treatments. You may be a good fit for this study. Please think about joining Duke researchers in a study that will lead to better treatments for millions of people living with obesity	The RESILIENCE study aims to help people who want to lose weight and improve their heart health. We want to understand why some people with weight issues develop heart disease while others do not. Likewise, we want to understand why some people respond to weight loss treatments while others do not. Participating in this study may make it possible for you to get better weight management and heart health treatments in the future. Please consider joining us in the RESILIENCE study!	Millions of people in the US are at risk for heart disease, and weight is an important part of this risk. We are doing the RESLIENCE Study to understand the differences in heart health between people who have obesity and those who do not. Although you are not overweight, by joining this study you can help us understand these differences. Please consider partnering with Duke researchers on a study that will lead to improved heart health for many people in the future.	We know that weight is an important part of heart health. However, you may be surprised to know that we do not yet fully understand how weight affects heart health. We are doing the RESILIENCE study to understand why some people with obesity develop heart disease while others do not. Although you are not overweight, by joining the study, you can help us discover an answer to this important scientific question. Please consider joining Duke researchers in the RESILIENCE study, and advancing science to prevent heart disease.

© 2026 Gouda P et al. *JAMA Network Open*.

eTable 2. Exploratory Subgroup Analysis of Primary Outcome of Potential Participant Engagement

	**Recruitment message**	**Interaction *P* value**	**Recruitment modality**	**Interaction *P* value**
	**Altruistic vs Individualistic relative risk (99% CI)**		**Email vs Patient portal relative risk (99% CI)**	
Age, y				
Age ≤60	1.21 (1.00-1.48)	.03	1.51 (1.24-1.85)	.04
Age >60	0.95 (0.77-1.16)		1.92 (1.55-2.38)	
Race				
White	1.08 (0.92-1.28)	.50	1.56 (1.32-1.85)	.06
Black or African American	1.02 (0.74-1.40)		1.97 (1.41-2.75)	
Other[a]	1.38 (0.76-2.51)		2.48 (1.29-4.75)	
Sex				
Male	1.11 (0.87-1.43)	.72	1.81 (1.39-2.36)	.38
Female	1.07 (0.90-1.27)		1.63 (1.37-1.94)	
Study phenotypes				
Obese low risk	0.96 (0.76-1.23)	.19	1.93 (1.49-2.49)	.03
Obese high risk	1.09 (0.86-1.37)		1.78 (1.40-2.27)	
Non-obese low risk	1.24 (0.95-1.63)		1.34 (1.02-1.75)	

[a]"Other" races included: American Indian or Alaska Native, Asian, more than one race and Native Hawaiian or Other Pacific Islander.
Robust log-linear regression model for the association of potential participant engagement and exposure to recruitment message and modality type adjusted for study phenotype.

© 2026 Gouda P et al. *JAMA Network Open*.
