# Supplementary material for: Messaging Modality and Content for Recruitment of Research Participants: A Randomized Clinical Trial
Source: JAMA Netw Open. 2026 May 22;9(5):e2614046. doi: 10.1001/jamanetworkopen.2026.14046 (PMC13197867; doi:10.1001/jamanetworkopen.2026.14046)
Supplement: Supplement 3. — Data Sharing Statement [file jamanetwopen-e2614046-s003.pdf]

Data Sharing Statement

Gouda. Messaging Modality and Content for Recruitment of Research Participants. *JAMA Netw Open*. Published May 22, 2026. doi:10.1001/jamanetworkopen.2026.14046

Data
Additional Information: NCT04551872
Data available: No
